# Elevating plant immunity by translational regulation of a rice WRKY transcription factor

**DOI:** 10.1111/pbi.14243

**Published:** 2023-11-23

**Authors:** Chao Zheng, Jie Zhou, Xiaoya Yuan, Ersong Zheng, Xiuli Liu, Weijun Cui, Chengqi Yan, Yueyan Wu, Wenyuan Ruan, Keke Yi, Jianping Chen, Xuming Wang

**Affiliations:** ^1^ State Key Laboratory for Managing Biotic and Chemical Threats to the Quality and Safety of Agroproducts, Ministry of Agriculture Key Laboratory for Plant Protection and Biotechnology, Zhejiang Provincial Key Laboratory of Plant Virology, Institute of Virology and Biotechnology Zhejiang Academy of Agricultural Sciences Hangzhou P. R. China; ^2^ College of Plant Protection Northwest A&F University Yangling P.R. China; ^3^ Institute of Biotechnology Ningbo Academy of Agricultural Sciences Ningbo P.R. China; ^4^ Zhejiang Wan Li University Ningbo P.R. China; ^5^ Institute of Agricultural Resources and Regional Planning Chinese Academy of Agricultural Sciences Beijing China; ^6^ Institute of Plant Virology Ningbo University Ningbo P. R. China

**Keywords:** OsWRKY7, alternative translation, protein stability, basal immunity, *Xoo*

## Abstract

Plants have intricate mechanisms that tailor their defence responses to pathogens. WRKY transcription factors play a pivotal role in plant immunity by regulating various defence signalling pathways. Many WRKY genes are transcriptionally activated upon pathogen attack, but how their functions are regulated after transcription remains elusive. Here, we show that *OsWRKY7* functions as a crucial positive regulator of rice basal immunity against *Xanthomonas oryzae* pv. *oryzae* (*Xoo*). The activity of OsWRKY7 was regulated at both translational and post‐translational levels. Two translational products of OsWRKY7 were generated by alternative initiation. The full‐length OsWRKY7 protein is normally degraded by the ubiquitin–proteasome system but was accumulated following elicitor or pathogen treatment, whereas the alternate product initiated from the downstream in‐frame start codon was stable. Both the full and alternate OsWRKY7 proteins have transcriptional activities in yeast and rice cells, and overexpression of each form enhanced resistance to *Xoo* infection. Furthermore, disruption of the main AUG in rice increased the endogenous translation of the alternate stabilized form of OsWRKY7 and enhanced bacterial blight resistance. This study provides insights into the coordination of alternative translation and protein stability in the regulation of plant growth and basal defence mediated by the OsWRKY7 transcription factor, and also suggests a promising strategy to breed disease‐resistant rice by translation initiation control.

## Introduction

Protein homeostasis is essential for cell viability. Various types of intricate mechanisms are coordinated to maintain the required amount and diversity of proteins in rapid response to environmental changes. During translation initiation, the selection of the AUG initiation codon is controlled by the scanning model conserved in eukaryotes, which is usually subject to the ‘first‐AUG rule’ (Hinnebusch, 2014; Kozak, 2002). However, alternative translation can sometimes be initiated at downstream AUG codons by context‐dependent leaky scanning and re‐initiation (Kozak, 2002). The flexibility of translation initiation control is versatile in determining both the efficiency and composition of protein translation (Meijer and Thomas, 2002).

In parallel to translational regulation, protein homeostasis is also sustained by the degradation pathways (Beese *et al*., 2020; Ciechanover, 2006; Hershko and Ciechanover, 1998; Varshavsky, 2019), which are crucial for timely disposal of unwanted proteins. In eukaryotic cells, the selective degradation of many short‐lived proteins is carried out through the ubiquitin proteasome system (UPS) (Hershko and Ciechanover, 1998; Vierstra, 2009). UPS‐mediated proteolysis regulates almost all of the intracellular processes of plant biology (Vierstra, 2009), and the importance of this pathway in plant–pathogen interactions has been increasingly highlighted (Dielen *et al*., 2010).

Plants maintain a dynamic balance between growth and defence in the face of continual challenges from a range of pathogens. Defence proteins are therefore under tight control to minimize the unnecessary fitness penalties associated with continuous activation of the defence response. The defence induction involves the recognition of microbe/damage‐associated molecular patterns (M/DAMPs) by host pattern‐recognizing receptors (PRRs) leading to pattern‐triggered immunity (PTI) or basal immunity in plants (Boller and Felix, 2009). The second type of defence is mounted by the detection of pathogen‐derived effectors by intracellular nucleotide‐binding and leucine‐rich repeat (NLR) immune receptors resulting in effector‐triggered immunity (ETI) (Araújo *et al*., 2019; Maekawa *et al*., 2011). Both PRR and NLR immune receptors are regulated by the plant UPS. In *Arabidopsis*, the FLAGELLIN receptor FLS2 (FLAGELLIN‐SENSING 2) is polyubiquitinated by PUB12/13 (Plant U‐Box 12/13) for FLAGELLIN‐induced turnover, thus attenuating immune signalling (Lu *et al*., 2011). Overaccumulation of SNC1, a Toll–interleukin 1 receptor (TIR)‐type NLR, leads to constitutive defence responses and consequent dwarfism (Zhang *et al*., 2003). The stability of SNC1 protein is controlled by the F‐box protein CPR1 for ubiquitination and degradation (Cheng *et al*., 2011). The *Arabidopsis* NPR1 (nonexpresser of *PR* genes 1) protein is a master immune regulator of systemic acquired resistance (SAR) (Fu and Dong, 2013). AtNPR1 is constantly degraded in the nucleus by the 26S proteasome which has dual roles in both preventing and stimulating gene transcription during SAR induction (Spoel *et al*., 2009). Overexpression of *AtNPR1* in rice enhanced disease resistance to multiple pathogens but had detrimental effects on plant growth (Fitzgerald *et al*., 2004; Quilis *et al*., 2008). Similarly, OsNPR1, one of the AtNPR1 orthologues in rice, is also regulated by ubiquitin‐mediated degradation through interaction with the Cullin 3 E3 ligase component (OsCUL3a), and accumulation of OsNPR1 in the *oscul3a* mutant causes cell death (Liu *et al*., 2017). *Arabidopsis TBF1*, a major molecular switch for growth‐to‐defence transition, is tightly regulated at both the transcriptional and translation levels. Translation of *TBF1* is normally suppressed by two uORFs within the 5′ leader sequence but promoted upon immune induction (Pajerowska‐Mukhtar *et al*., 2012). This unique regulatory mechanism, uORF‐mediated translation inhibition, was successfully used to engineer disease‐resistant plants without fitness costs (Xu *et al*., 2017).

The WRKY gene family is a large group of transcription factors that play important roles in regulation of defence responses in plants (Pandey and Somssich, 2009). WRKY proteins participate in transcriptional reprogramming by binding to W‐box elements in target promoters during a variety of immune responses including PTI, ETI, and SAR (Eulgem, 2005; Eulgem and Somssich, 2007; Maleck *et al*., 2000). Many recent studies have explored in detail the roles and signalling of WRKYs in regulation of stress responses (Chen *et al*., 2019; Phukan *et al*., 2016; Wani *et al*., 2021), but there is very limited knowledge of the mechanisms by which plants dynamically regulate WRKY protein homeostasis to adapt to their environment (Phukan *et al*., 2016). It has been shown that OsWRKY45, a pivotal regulator in SA/BTH‐induced disease resistance to both fungal blast (Shimono *et al*., 2007) and bacterial leaf blight (Shimono *et al*., 2012) in rice, is degraded in the nucleus through the ubiquitin–proteasome system to prevent spurious defence activation in the absence of pathogen attack (Matsushita *et al*., 2013). AtWRKY53, which positively regulates leaf senescence, is targeted by the HECT domain E3 ubiquitin ligase UPL5 for its polyubiquitination and degradation, to ensure correct timing of senescence induction (Miao and Zentgraf, 2010).

Prior studies have established that *OsWRKY7* expression can be triggered by pathogen stress (Ryu *et al*., 2006) and that its overexpression confers resistance to blast fungus (Tun *et al*., 2023). In this study, we conducted a comprehensive functional characterization of *OsWRKY7*, demonstrating its positive role in mediating basal immunity against the bacterial pathogen *Xoo*. In addition to transcriptional regulation, OsWRKY7 was also tightly regulated at the protein level. Alternative translation from both the main open reading frame (mORF) and downstream in‐frame ORF (diORF) of *OsWRKY7* generated two isoforms with different protein stabilities. The full‐length OsWRKY7 was polyubiquitinated and constitutively degraded through the 26S proteasome pathway. Treatment with the bacterial elicitor Flg22 and *Xoo* increased the protein level of OsWRKY7. The domain essential for degradation was located at the N‐terminus and was different from the domains responsible for transcriptional activation and subcellular localization. The alternative translated protein lacked the degradation region and was therefore stable and functional. Overexpression of the full‐length or the short stable isoform enhanced bacterial blight resistance in rice. Similar to the upstream open reading frame (uORF), the mORF of *OsWRKY7* represses the translation of the diORF. Translation disrupting of the mORF by genome editing results in increased protein expression of the diORF and enhanced disease resistance to *Xoo* through increased *PR* transcript accumulation and ROS production. In addition, we were interested to find that proteasomal degradation and alternative translation were also features of several *WRKY* genes in the same subclade as *OsWRKY7*. Our results suggest that the production of appropriate amounts of OsWRKY7 protein is essential for normal growth and effective basal defence. Translational regulation could be explored as a route to optimize the production of defence proteins for breeding of disease‐resistant crops with less fitness cost.

## Results

### *OsWRKY7* is a positive regulator of rice basal defence against bacterial blight

**Figure 1:**
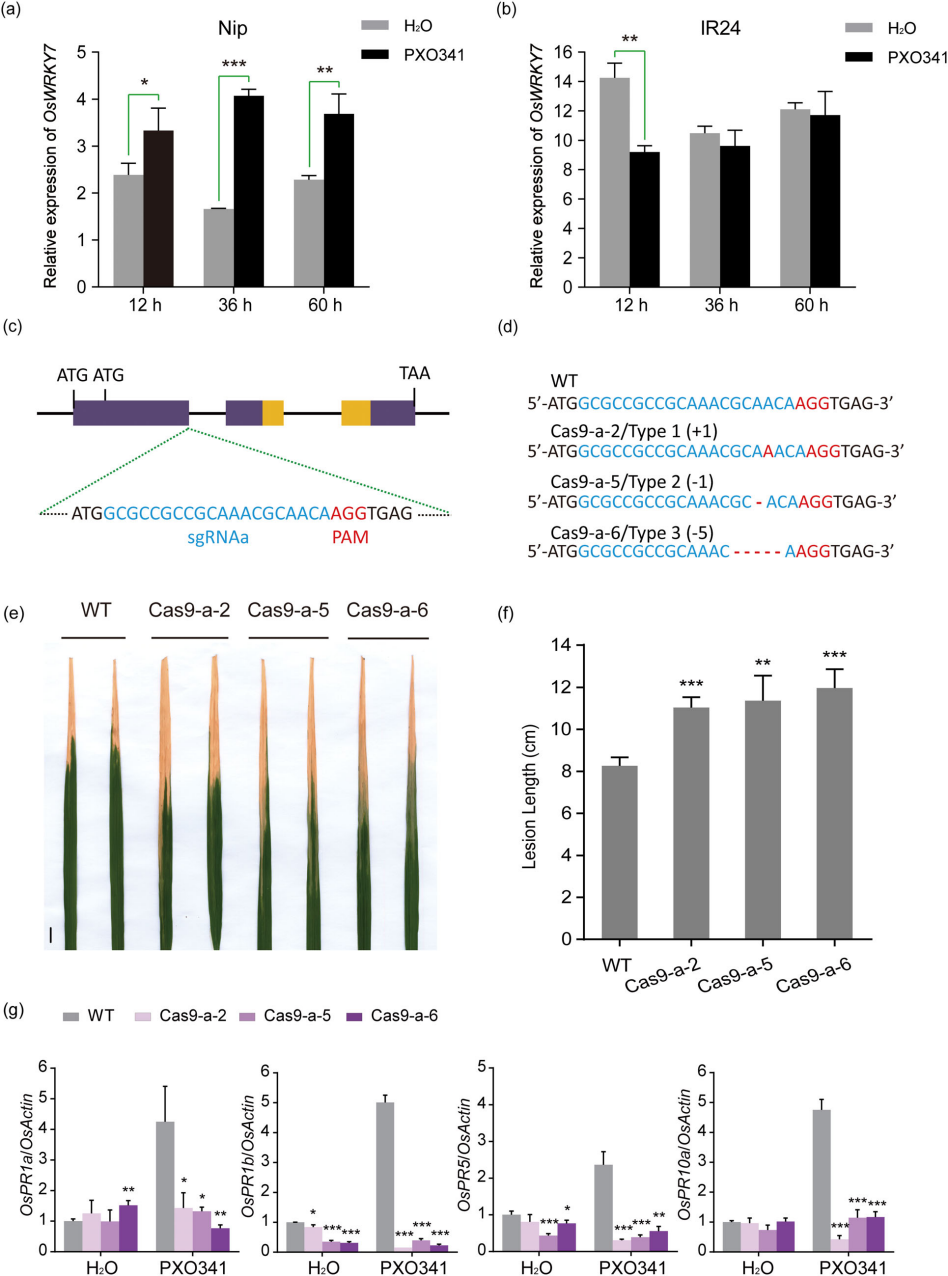
*OsWRKY7* loss of function mutant plants have increased susceptibility to *Xoo* infection. (a) Expression profile of *OsWRKY7* in *Nipponbare* (Nip) inoculated with *Xoo* strain PXO341 or H_2_O for the time point indicated. (b) Expression profile of *OsWRKY7* in IR24 inoculated with *Xoo* strain PXO341 or H_2_O. (c) Gene structure of *OsWRKY7*. The WRKY domain resides in the second and third exons are shown in yellow. The sgRNAa target sequence (blue letters) and PAM site (red letters) are shown at the end of the first exon. (d) Representative types of mutant alleles identified at the sgRNAa target. The wild‐type sequence is shown at the top and mutant lines (Cas9‐a‐2, 5, 6) with three types of mutation are shown below. The numbers of deletions or insertions are shown in brackets. The red letters indicate the PAM site. (e) Representative leaves with typical lesions at 14 dpi were shown from the WT and three homozygous Cas9‐a lines. Scale bar: 1 cm. (f) Lesion lengths on the leaves of the WT and Cas9‐a lines inoculated in (e) at 14 dpi. Bars represent mean lesion lengths±SD (*n* ≥ 3). Significant differences between WT and mutation lines are indicated as **P < 0.01, ***P < 0.001 by Student's *t*‐test. (g) qRT‐PCR analysis of *OsPR1a*, *OsPR1b*, *OsPR5*, and *OsPR10a* expression in WT and Cas9‐a mutation lines challenged with PXO341 or H_2_O for 48 h. Data are shown as means ± SD (*n* = 3) of the fold change relative to the level of WT with H_2_O. Statistically significant differences to the WT control are indicated as *P < 0.05, **P < 0.01, and ***P < 0.001 by Student's *t*‐test.

In an earlier study of the expression of the *WRKY* gene superfamily in rice, *OsWRKY7* increased rapidly during an incompatible interaction between rice and the bacterial blight pathogen *Xanthomonas oryzae* pv. o*ryzae* (*Xoo*) (Ryu *et al*., 2006). To confirm its possible role in the rice defence response to bacterial blight, we investigated the expression of *OsWRKY7* in compatible rice varieties infected with *Xoo* strain PXO341 by qRT‐PCR. Compared to its basal expression in H_2_O‐treated japonica cultivar *Nipponbare* (Nip), *OsWRKY7* was increased following pathogen inoculation at the time point 12, 36, and 60 h (Figure 1a). However, *OsWRKY7* was not induced by PXO341 in IR24 (Figure 1b), which is a susceptible near‐isogenic parent of the IRBB lines which have one or more bacterial blight resistance (*Xa*) genes (Huang *et al*., 1997). These results suggest that *OsWRKY7* may be involved in the basal immune response in rice to *Xoo* infection.

To characterize the function of *OsWRKY7* in regulation of bacterial blight resistance, we generated loss‐of‐function mutants of *OsWRKY7* using CRISPR/Cas9 technique in the Nip background (Ma *et al*., 2015). Two single‐guide RNAs (sgRNAa and sgRNAb) were designed within the first exon (Figure 1c; Figure S2a). Based on PCR and sequencing analysis (Figures S1a and S2b), three different mutations were identified in plants targeted by sgRNAa and sgRNAb, respectively (Figure 1d; Figure S2b), causing frameshift expression of the OsWRKY7 protein (Figures S1b and S2c). Three homozygous mutant lines at the sgRNAa target (*oswrky7‐Cas9‐a*) were selected and inoculated with *Xoo* at the booting stage. Compared to WT plants, the mutants had more severe disease symptoms with longer lesions at 14 dpi (Figure 1e,f and Figure S1c). Consistently, the transcript levels of the pathogenesis‐related (PR) genes *PR1a*, *PR1b*, *PR5*, and *PR10a* were decreased in the mutant plants and their responses to *Xoo* infection were greatly impaired (Figure 1f). In addition, the *oswrky7‐Cas9‐b*‐mutant lines were also more susceptible than the controls to *Xoo* (Figure S2d). These results indicate that *OsWRKY7* plays a positive role in basal resistance against bacterial blight.

### OsWRKY7 protein undergoes alternative initiation from a downstream in‐frame ORF (diORF)

**Figure 2:**
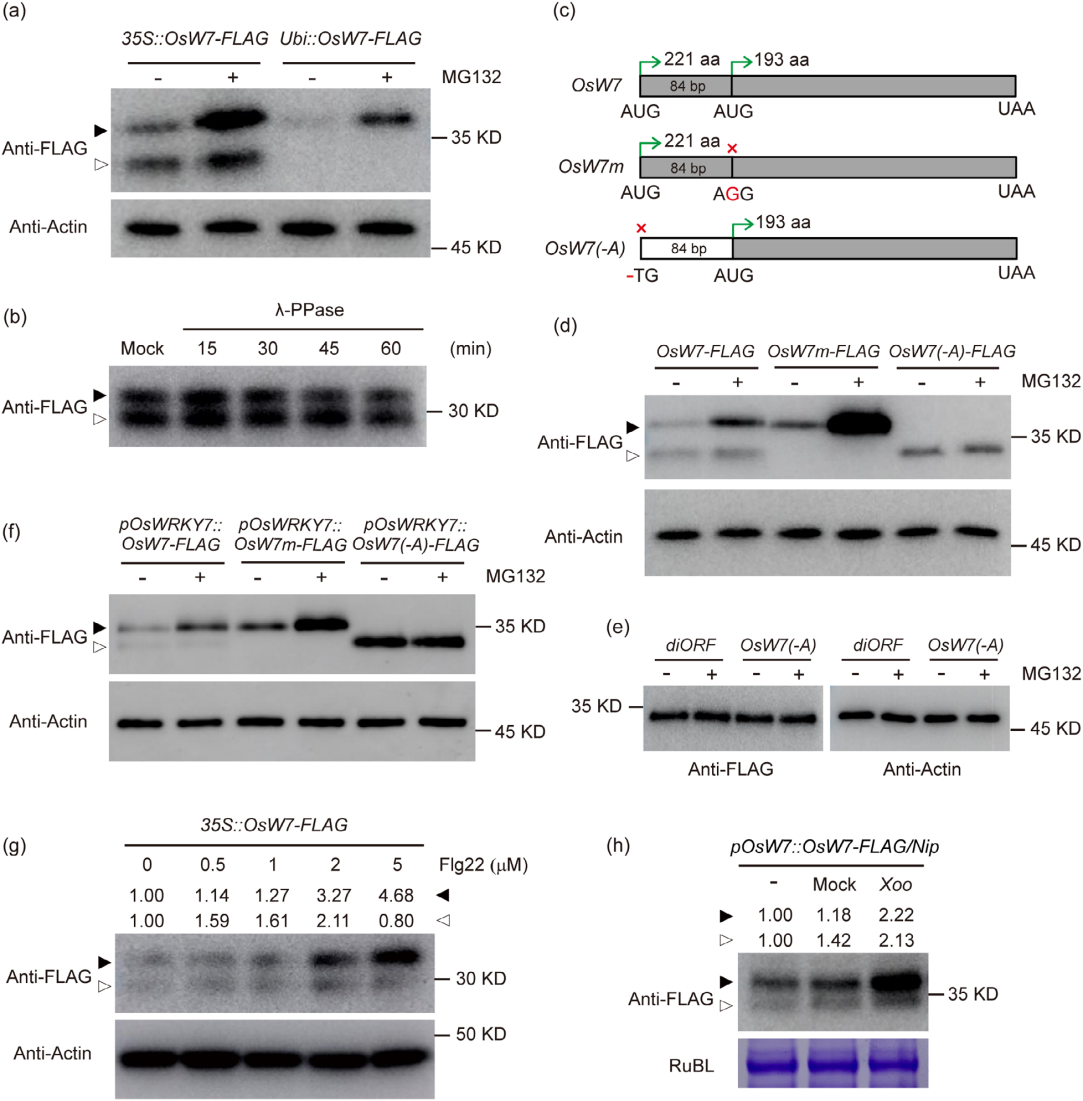
Generation of two OsWRKY7 protein isoforms by alternative translation initiation at two in‐frame AUG codons. (a) Comparison of the 3 × FLAG fused OsWRKY7 proteins expressed by the 35S or Ubiquitin promoter. Protoplasts were treated with DMSO (−) or 20 μM MG132 (+) for 4 h. Total protein was detected with anti‐FLAG antibody. The level of actin protein was used as an internal loading control. (b) *35S::OsWRKY7‐3 × FLAG* was transiently expressed in rice protoplasts and treated with λ‐PPase for different times as indicated. A sample in λ‐PPase buffer without phosphatase was used as mock control. (c) Schematic diagrams showing the coding sequence of *OsWRKY7* (*OsW7*) and derived sequences with modifications. The main and the second in‐frame AUG codons are indicated. The green arrowheads indicate translation of the corresponding proteins of 221 and 193 amino acids respectively. The *OsW7m* represents the coding sequence with a point mutation of the second AUG to AGG, and the *OsW7(−A)* represents a deletion of the first AUG to UG. The red crosses indicate the disabled translation of the corresponding proteins of 221 or 193 amino acid respectively. (d) The three types of coding sequences indicated in (b) were fused with 3 × FLAG and transiently expressed in rice protoplasts under control of the 35S promoter. The protoplasts were treated with DMSO (−) or 20 μM MG132 (+). (e) The *OsW7(−A)* coding sequence and the second *diORF* were fused with 3xFLAG and transiently expressed in rice protoplasts under control of the 35S promoter. The protoplasts were treated with DMSO (−) or 20 μM MG132 (+). (f) The *OsW7*, *OsW7m*, and *OsW7(−A)* genomic sequences were transiently expressed in rice protoplasts under control of the native *OsWRKY7* promoter. Protoplasts were treated with DMSO (−) or 20 μM MG132 (+). (g) Pathogen mimic treatment of protoplasts transfected with *35S::OsWRKY7‐3xFLAG*. 0 μM, 0.5 μM, 1 μM, 2 μM, and 5 μM Flg22 were added to the protoplasts and treated for 2 h. Total protein was extracted and detected with anti‐FLAG antibody. The level of actin protein was used as an internal loading control. (h) PXO341 treatment of *OsWRKY7* promoter regulated *OsWRKY7‐FLAG* transgenic plants. Total proteins were extracted from leaves after treatment with H_2_O + 0.05% Silwet L‐77 (Mock), PXO341 + 0.05% Silwet L‐77 (*Xoo*), or without treatment (−). Signals were detected with anti‐FLAG antibody. Coomassie blue‐stained Rubisco large protein (RubL) was used as loading control. The black and white arrowheads indicate the full length and alternative translated OsWRKY7 proteins respectively. Relative protein abundance in (g) and (h) were calculated to the control by ImageJ.

To further investigate the role of OsWRKY7 in basal defence, we constructed the *35S::OsWRKY7‐FLAG* vector and transfected rice protoplasts for protein expression analysis. Surprisingly, two close protein bands were detected in immunoblots, and the upper band was significantly increased when incubated with MG132, a 26S proteasome inhibitor (Figure 2a). Since many WRKY proteins have been reported to be phosphorylated by specific protein kinases for the regulation of plant immunity and stress adaptation (Chen *et al*., 2019), the potential phosphorylation of OsWRKY7 was examined via λ‐phosphatase (λ‐PPase) treatment. Notably, the two bands were insensitive to λ‐PPase treatment (Figure 2b), indicating that the upper band did not correspond to phosphorylated OsWRKY7‐FLAG. By comparing with the single protein expressed from the *Ubi::OsWRKY7‐FLAG* vector, the upper band was supposed to be the full‐length OsWRKY7 (Figure 2a).

Since the mRNA of *OsWRKY7* gene has no alternative splicing according to the analysis of the RNA‐seq data in NCBI (Figure S3), the two proteins are unlikely to be regulated by different splicing events. We then noticed the full‐length *OsWRKY7* CDS contains a second in‐frame start codon 84 bp downstream. The resulting 28 amino acid is about 2.7 kD, which closely matches the difference between the two bands. This suggests that the additional lower band might be an N‐terminal‐truncated protein, possibly produced by alternative translation of the diORF. To test this notion, two mutated constructs were generated to disable the main AUG by removing A (*OsW7(−A)*) and convert the second in‐frame AUG to AGG (*OsW7m*) respectively (Figure 2c). Then, these constructs were transiently expressed in rice protoplasts and treated with MG132. Immunoblots showed that inactivation of the main initiation site led to the production of the stable short protein only (Figure 2d), which size corresponded to the protein translated from the diORF (Figure 2e), whereas mutation of the second in‐frame AUG site simply resulted in the translation of the unstable full‐length protein (Figure 2d). To further elucidate whether the access to the second in‐frame AUG of *OsWRKY7* depends on the 35S promoter, a 3‐kb native promoter upstream from the main AUG was used to express the full‐length *OsWRKY7* gene in protoplasts. Apparently, two isoforms of OsWRKY7 were also produced with different stabilities (Figure 2f), whereas only one isoform remained when the main or second AUG was disrupted (Figure 2f). Besides, in protoplasts, two isoforms could be detected in transgenic plants regulated by the native promoter (Figures 2h and 6c) suggesting that the alternative translation of *OsWRKY7* occurs *in planta*.

Finally, we performed LC–MS/MS analysis to confirm whether the two bands were alternatively translated from *OsWRKY7*. In order to identify the peptide at the N‐terminal of the full‐length OsWRKY7, three Ser (S) residues at site 16, 28, and 43 were changed to Arg (R) for enzyme digestion. The mutated *OsWRKY7* (*OsWRKY7‐SR*) gene under control of the 35S promoter also expressed two protein bands which were obvious after MG132 treatment (Figure S4a). The upper and lower bands in MG132 or DMSO treatment were sent for LC–MS/MS analysis (Figure S4b). Peptides that targeted to the OsWRKY7‐SR protein were identified from all the bands (Table S3), but only the upper band contained the N‐terminal peptide after the first Met (Figure S4c), while the lower bands from both DMSO and MG132 treatment contain the peptides after the second Met (Figure S4c). Therefore, our results confirm the presence of two translational products of OsWRKY7 by dual initiation from two in‐frame start codons.

### The full‐length OsWRKY7 protein is degraded by the ubiquitin–proteasome pathway

**Figure 3:**
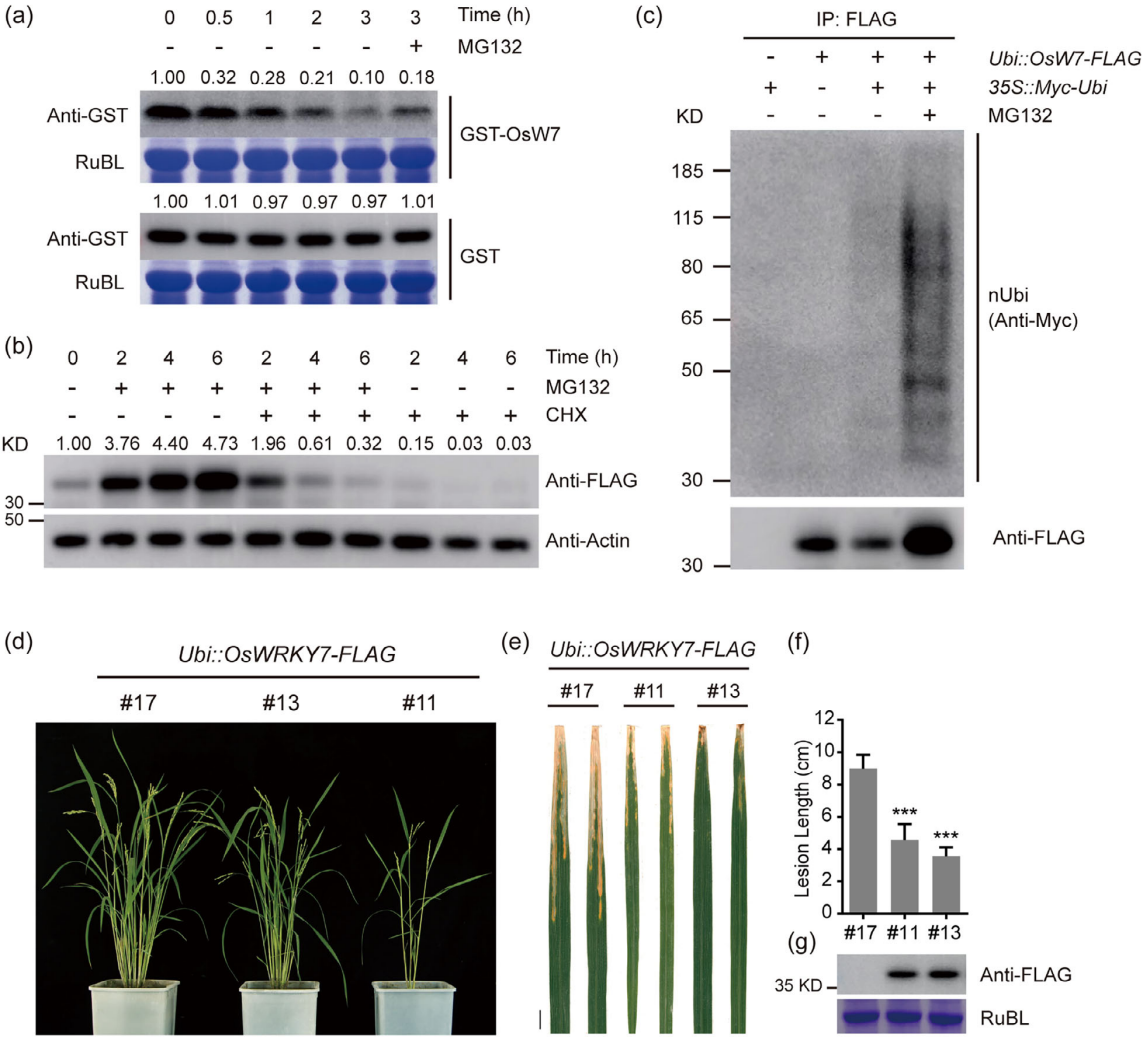
The full‐length OsWRKY7 protein is a positive regulator against *Xoo* and is degraded by the proteasome‐mediated pathway both in vivo and in vitro. (a) Cell‐free degradation assay of GST fused OsWRKY7 in wild‐type plant extracts treated without (−) or with (+) MG132 (100 μM) for indicated time. GST protein was used as non‐degraded control. RubL was used as loading control. The protein levels were calculated by ImageJ and the relative abundance at 0 h was set to 1.00. (b) *Ubi::OsWRKY7–3 × FLAG* was transiently expressed in rice protoplasts and treated with 20 μM MG132 and/or 50 μM CHX for 2 h, 4 h, and 6 h. Total protein was extracted and detected with anti‐FLAG and anti‐actin antibodies. The protein ratio of OsWRKY7‐FLAG to actin was calculated by ImageJ and the relative abundance in mock treatment was set to 1.00. (c) Ubiquitination of the full‐length OsWRKY7 in vivo. *Ubi::OsWRKY7‐FLAG* was transiently co‐expressed with or without *35S::Myc‐Ubi* in rice protoplasts treated with or without MG132 (50 μM). Polyubiquitinated OsWRKY7‐FLAG (indicated as nUbi) was detected using anti‐Myc antibody following immunoprecipitation with anti‐FLAG magnetic beads. The levels of immunoprecipitated (IP) OsWRKY7 proteins were detected with anti‐FLAG antibody. (d) Growth phenotype of *Ubi::OsWRKY7‐FLAG* transgenic lines in filling stage without *Xoo* infection. (e) Lesions on leaves of transgenic lines after PXO341 infection (17 dpi). Scale bar: 1 cm. (f) Lesion lengths on the leaves of transgenic lines at 17 dpi. Bars represent mean lesion lengths±SD (*n* ≥ 3). Significant differences between line #17 and other two lines are indicated as ***P < 0.001 by Student's *t*‐test. (g) OsWRKY7‐FLAG expression in transgenic lines. Total proteins were extracted from leaves and detected with anti‐FLAG antibody. RubL was used as loading control.

To further examine the stability of the full‐length OsWRKY7 protein, we performed cell‐free degradation analysis. Immunoblot analysis indicated that the recombinant GST‐OsWRKY7 protein was significantly decreased after 3 h incubation (Figure 3a). The addition of MG132 partially inhibited the degradation (Figure 3a). Then, we transiently expressed *Ubi::OsWRKY7‐FLAG* in rice protoplasts, in which the full‐length OsWRKY7 was dominantly translated (Figure 2a). The low amount of OsWRKY7 in the mock was gradually increased after prolonged MG132 treatment. In the co‐treatment with the protein synthesis inhibitor CHX, the protein amount gradually decreased to the lower level and further decreased in the treatment with CHX alone (Figure 3b), whereas the level of endogenous actin protein was constant (Figure 3b). In contrast, leupeptin and E‐64, the two different cysteine protease inhibitors of lysosomal degradation, did not prevent degradation of OsWRKY7 (Figure S5), suggesting that the 26S proteasome pathway is involved in the degradation of OsWRKY7. To support this idea, protein extracts from the rice protoplasts expressing *Ubi::OsWRKY7‐FLAG* with or without *35S::Myc‐Ubi* were immunoprecipitated and then detected by anti‐Myc and anti‐ubiquitin antibodies. As shown in Figure 3c and Figure S6, the level of polyubiquitinated OsWRKY7‐FLAG, indicated as a smearing ladder of bands, was greatly increased in protoplasts treated with MG132 compared to the mock treatment. These results demonstrate that OsWRKY7 is a fast‐turnover protein which is degraded via the ubiquitin/26S proteasome pathway.

### Over‐accumulation of the full‐length OsWRKY7 enhances disease resistance but affects plant growth

Since the full‐length OsWRKY7 protein is unstable under the normal condition, we analysed the protein level after elicitor/pathogen treatment. By transient expression of both the full‐length and short OsWRKY7 proteins under the 35S promoter, we found that the full‐length protein was increased more than the short form after Flg22 treatment (Figure 2g), suggesting pathogen mimic treatment could stimulate the full‐length protein accumulation. We also observed the same trends in transgenic plants under its native promoter after *Xoo* treatment (Figure 2h). These data indicated that alteration in protein levels plays an important role upon pathogen infection.

Then, we transformed plants with *Ubi::OsWRKY7‐FLAG* to overexpress the full‐length protein only. Finally, we obtained two lines with overexpressed protein levels (Figure 3g). And they showed resistance to *Xoo* infection as the lesion lengths were shorter than the control plant with no protein expression (Figure 3e,f). On the other hand, we observed impaired growth phenotype, especially line #11 (Figure 3d). So we concluded that the full‐length *OsWRKY7* positively regulates rice basal defence against *Xoo* but represses plant growth, implying a trade‐off effect between growth and defence response.

### Translation from the diORF stabilizes OsWRKY7 protein without eliminating the transcriptional activity or changing its subcellular localization

In the MG132 and CHX time course treatment, we found that the short OsWRKY7 protein translated from the diORF was stable in all the treatments (Figure S7), suggesting that the stability of OsWRKY7 was differentially regulated by alternative translation, and the N‐terminal region before the second Met (28 amino acid) is essential for degradation.

**Figure 4:**
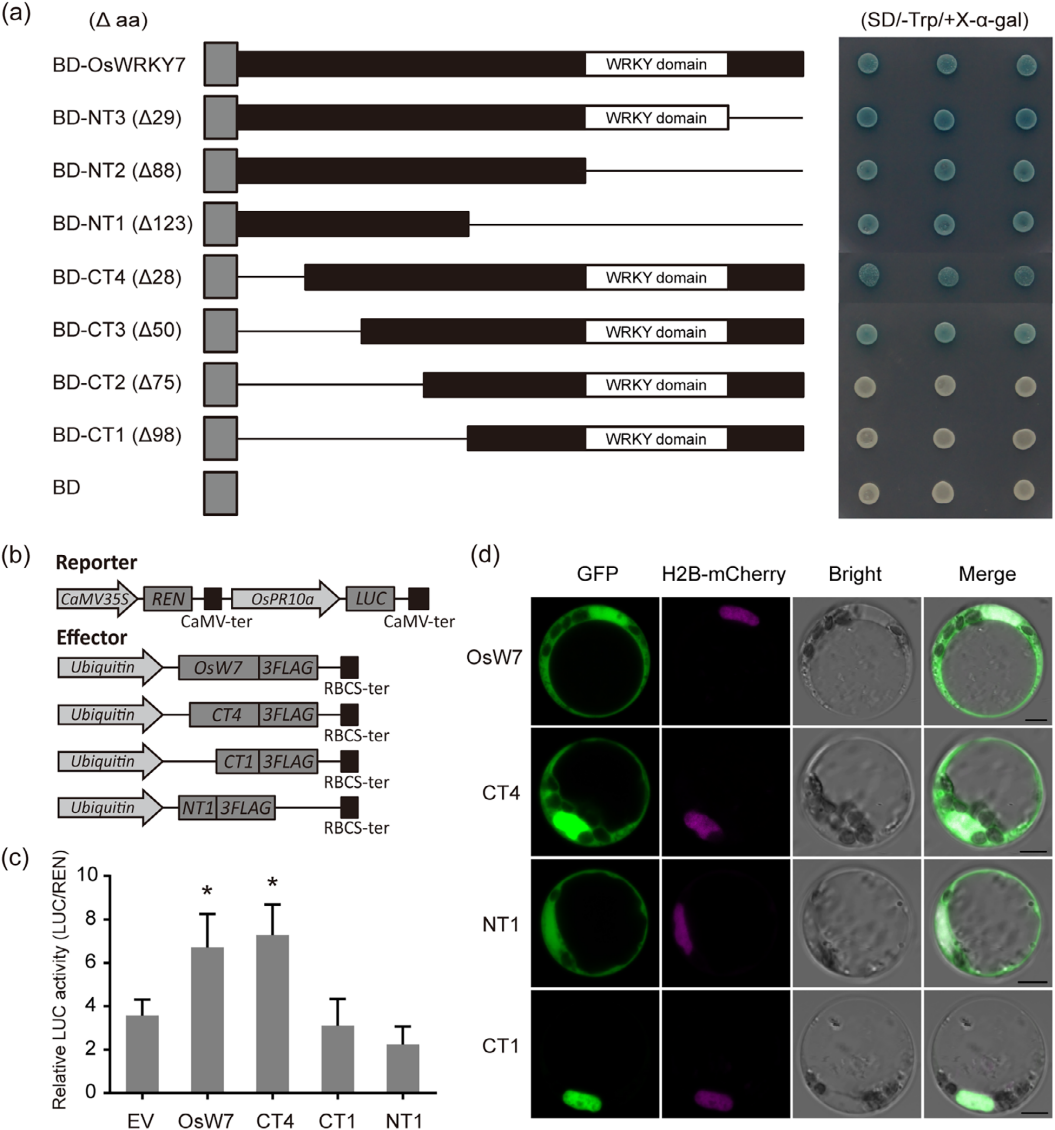
The region responsible for OsWRKY7 degradation is different to the transactivation domain and irrelevant to its localization. (a) The transcriptional activity of different N‐terminal or C‐terminal truncated proteins in yeast cells. The number of deleted amino acids is given in brackets for each truncated fragment. Single lines represent the removed fragments (b) Schematic diagram of the effector and reporter vectors used for dual luciferase reporter assay. The reporter vector contained a 2523 bp *OsPR10a* promoter before the firefly luciferase (LUC). The effector vector contained the full‐length OsWRKY7 (OsW7) protein and NT1, CT3, and CT4 truncations expressed under the *Ubi* promoter. (c) Transactivation assay of the *OsPR10a*‐*LUC* reporter by the effectors indicated in (b). Relative LUC activities were expressed by normalizing the LUC signals to the value of REN. Data are means ± SD (*n* = 3). The empty effector vector (EV) serves as the control. Significant differences are indicated as *P < 0.05 by Student's *t*‐test. (d)  Subcellular localization of the full‐length OsWRKY7 (OsW7) protein and its truncations (NT1, CT3, and CT4) which were fused with GFP in rice protoplasts. A H_2_B‐mCherry vector was co‐transformed together to indicate the nucleus. Scale bars: 4 μM.

To explore whether the domain required for degradation is also indispensable to drive the full transcriptional activity as reported in some cases (Matsushita *et al*., 2013; Muratani and Tansey, 2003), a number of deletion mutants of the OsWRKY7 protein were fused to the GAL4 DNA binding domain (BD) and tested the reporter activation in yeast. As shown in Figure 4a, the shortest N‐terminal part (NT1), which lacked the entire WRKY domain, could still activate the *MEL1* reporter, whereas the remaining C‐terminal part (CT1) had no transactivation activity. By additional N‐terminal deletions (CT2–CT4), we found that deletion of the 28 or 50 amino acids at the N‐terminus (CT4 and CT3) did not affect the transcriptional activity, but a further deletion of 75 amino acids (CT2) thoroughly impaired it. These results suggest that the activation domain of OsWRKY7 is located in the 51–75 amino acid region and is different to the N‐terminal region required for degradation.

Subsequently, we tested the activity of the deletion proteins on the transcriptional regulation of pathogenesis‐related genes in plants. The effector constructs containing the full‐length *OsWRKY7* or the deletion fragments (NT1, CT1, and CT4) driven by the ubiquitin promoter were co‐expressed with the *OsPR10a‐pro::LUC* reporter construct in rice protoplasts (Figure 4b). The results showed that both the full‐length OsWRKY7 (OsW7) and the N‐terminal truncated protein translated from the second AUG (CT4) could activate the reporter gene expression (Figure 4c). By contrast, the proteins consisting only the activation domain (NT1) or the WRKY DNA‐binding domain (CT1) were unable to activate the *OsPR10a* promoter (Figure 4c). Together, these results indicate that both the activation and WRKY domains, but not the degradation domain, are involved in the OsWRKY7‐mediated transcriptional regulation.

We then investigated the functional relevance of the subcellular localization to the degradation of OsWRKY7. GFP fluorescences of both the full‐length OsWRKY7 and the CT4 protein without the N‐terminal degradation domain were predominantly detected in the nucleus but with a weak cytoplasmic signal (Figure 4d), whereas the CT1 protein containing the WRKY domain was confined to the nucleus and the NT1 without the WRKY domain was evenly distributed in both the cytoplasm and nucleus (Figure 4d). These results demonstrate that the degradation domain does not overlap with the signal peptides for nuclear and cytoplasmic localization of OsWRKY7.

### Overexpression of the stabilized OsWRKY7 encoded from the diORF confers enhanced resistance to bacterial blight

**Figure 5:**
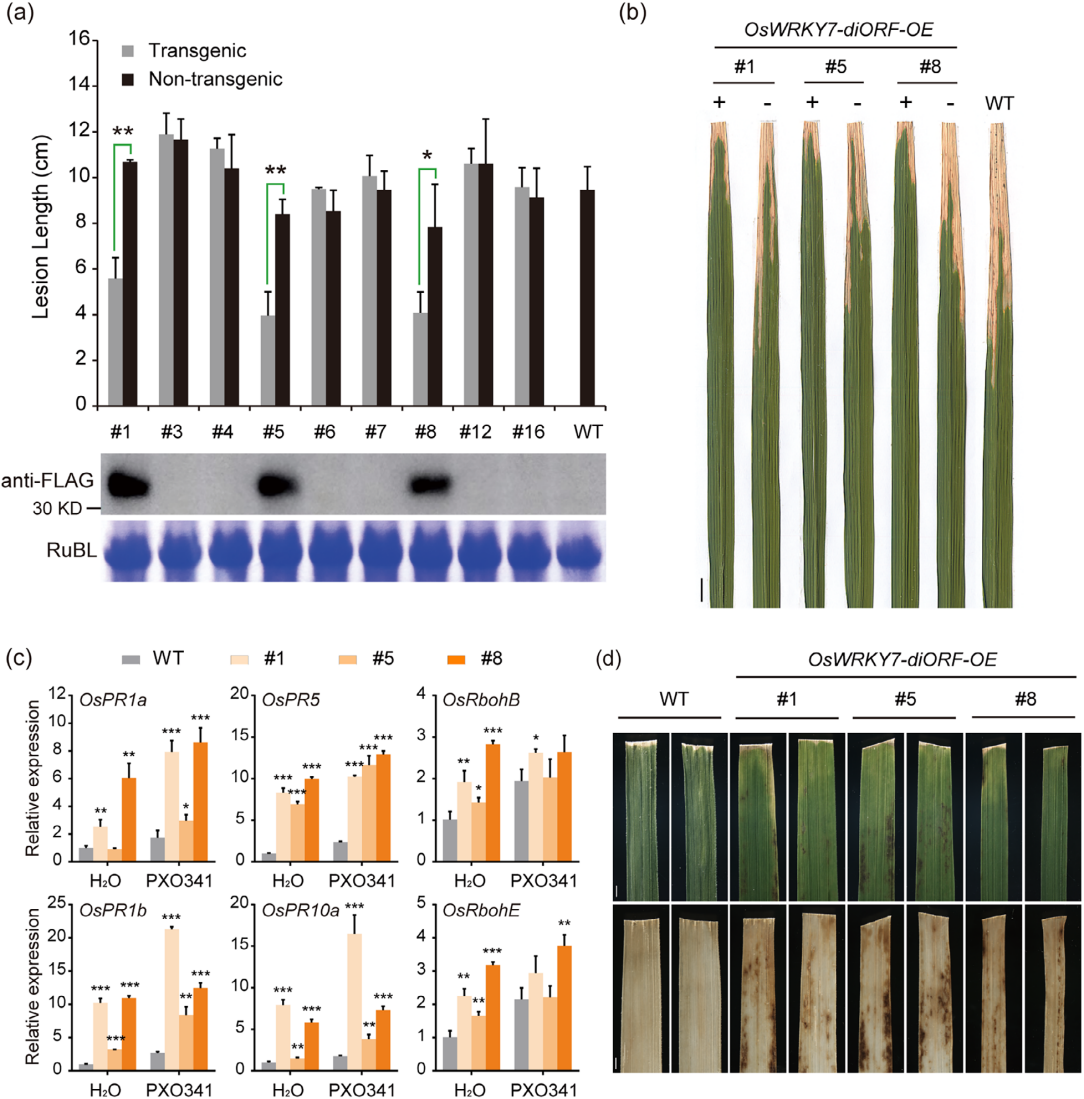
Overexpression of the stabilized OsWRKY7 from the diORF confers enhanced resistance to *Xoo* infection. (a) Lesion lengths on leaves of *OsWRKY7‐diORF‐OE* transgenic lines and wild type (WT) after *Xoo* (PXO341) infection for 14 days. Segregated T_1_ plants with transgene (grey bar) or without transgene (black bar) were identified by PCR analysis before inoculation. Bars represent mean lesion lengths±SD (*n* ≥ 3). Statistical analysis was performed by Student's *t*‐test (*P < 0.05, **P < 0.01). OsWRKY7‐diORF protein was detected with anti‐FLAG antibody. RubL was used as loading control. (b) Representative leaves with lesions from three T_1_ *diORF‐OE* lines and WT after PXO341 infection for 14 days. Segregated T_1_ plants with (+) or without (−) transgene were determined by PCR amplification. Scale bar: 1 cm. (c) qRT‐PCR analysis of *OsPR1a*, *OsPR1b*, *OsPR5*, *OsPR10a*, *OsRbohB*, and *OsRbohE* expression in WT and *diORF‐OE* lines challenged with *Xoo* or H_2_O for 48 h. Data are shown as means ± SD (*n* = 3) of the fold change relative to the levels in WT with H_2_O after normalization to the *OsActin* gene. Significant differences to the WT controls are indicated as *P < 0.05, **P < 0.01, and ***P < 0.001 by Student's *t*‐test. (d) Estimation of H_2_O_2_ levels in leaves of WT and *diORF‐OE* lines. Leaves inoculated with *Xoo* at 5 dpi (upper panels) were stained with 3,3′‐diaminobenzidine (DAB) and photographed after decolouring (lower panels). Scale bars: 1 mm. Two leaves of each line are shown.

**Figure 6:**
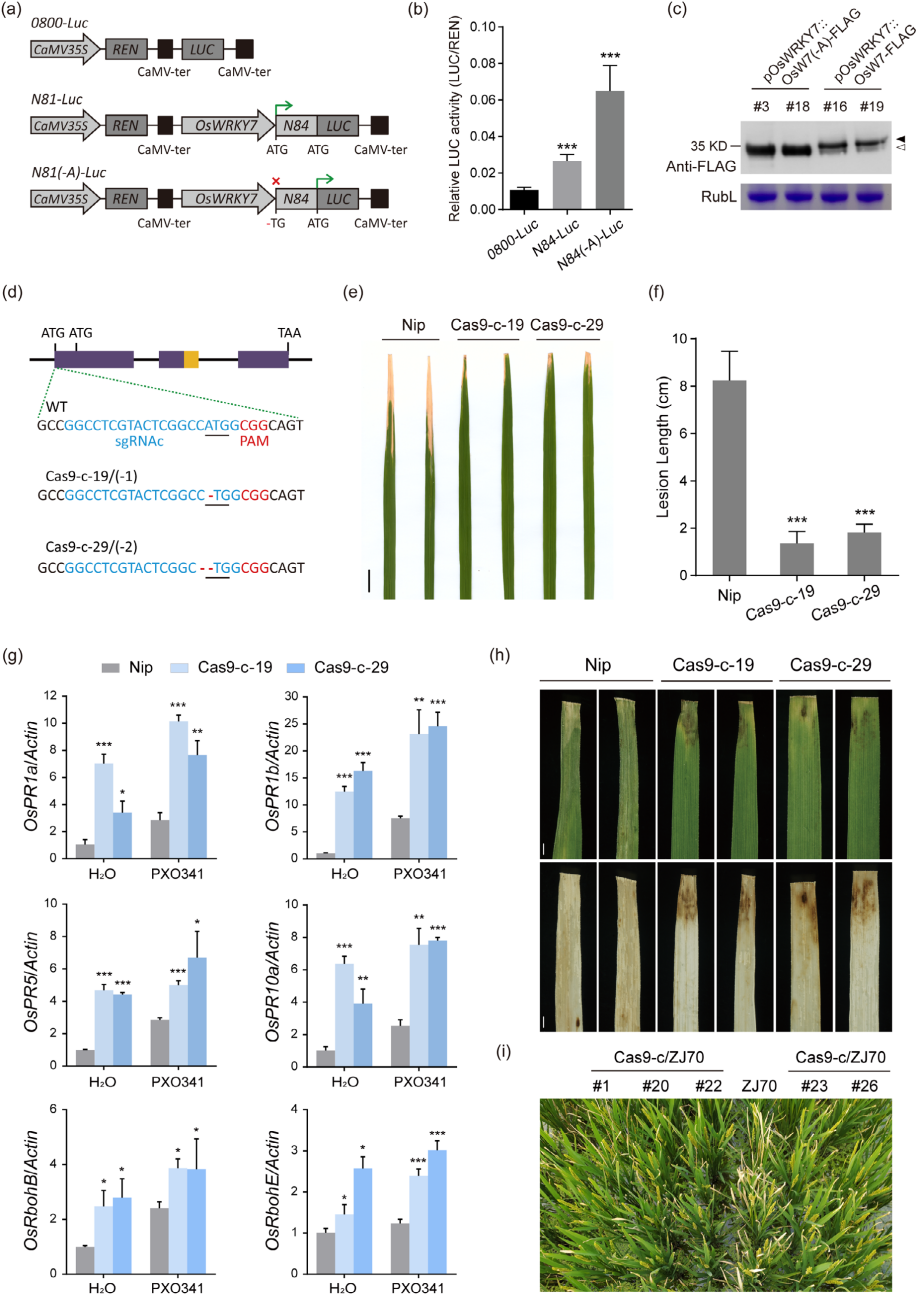
Translation disrupting of the mORF results in increased protein expression of the diORF and enhanced disease resistance to *Xoo*. (a) Schematic diagram of the reporter vectors used for dual luciferase reporter assay in (b). *OsWRKY7* promoter and the N terminal 84 bp were infused with the luciferase reporter gene (Luc). The *N84‐(−A)‐Luc* vector was similar to *N84‐Luc* except the A of main ATG was deleted. (b) Relative LUC activity of vectors in (a) after transient expressed in rice protoplasts. (c) Compare the protein levels in plants transformed with *OsWRKY7(−A)‐FLAG* and *OsWRKY7‐FLAG* under the native promoter. Two lines with highest protein expression in each transgenic population were used. Total proteins were extracted from leaves and detected with anti‐FLAG antibody. RubL was used as loading control. (d) The sgRNAc target sequence (blue letters) and the PAM site (red letters) shown include the main ATG (underlined). Representative types of mutant alleles were identified at the sgRNAc target. The WT sequence is shown at the top and mutant lines (Cas9‐c‐19, 29) with different mutations shown below. The numbers of deletions are shown in brackets for each type. (e) Leaves with typical lesions from the Nip and two homozygous Cas9‐c mutant lines after PXO341 infection (14 dpi). Scale bar: 2 cm. (f) Lesion lengths on leaves of Nip and mutant lines inoculated in (e) at 14 dpi. Bars represent mean lesion lengths±SD (*n* ≥ 3). Statistical analyses were performed by Student's *t*‐test between mutant lines and WT (***P < 0.001). (g) qRT‐PCR analysis of *OsPR1a*, *OsPR1b*, *OsPR5*, *OsPR10a*, *OsRbohB*, and expression in Nip and Cas9‐c mutation lines challenged with *Xoo* or H_2_O for 48 h. Data are shown as means ± SD (*n* = 3) as the fold changes relative to the level in the Nip with H_2_O after normalization to the *OsActin* gene. Significant differences to the Nip controls are indicated as *P < 0.05, **P < 0.01, and ***P < 0.001 by Student's *t*‐test. (h) H_2_O_2_ levels in leaves of Cas9‐c mutation lines and Nip. Leaves inoculated with *Xoo* at 5 dpi (upper panels). The same *Xoo*‐infected leaves were stained with 3,3′‐diaminobenzidine (DAB) and photographed after decolouring (lower panels). Scale bars: 1 mm. (i) *Xoo*‐resistant phenotype of Cas9‐c‐mutant lines in ZJ70 background. Picture was taken in patch field 17 days after PXO341 infection.

Since the alternative translation of OsWRKY7 initiated from the diORF occurred under its native promoter (Figures 2h and 6c), and this N‐terminal‐truncated protein retained the full active transcriptional activity and normal cellular distribution in protoplasts (Figure 4c,d), we further investigated its potential function in the regulation of rice resistance to bacterial blight. Transgenic plants with overexpression of the diORF (*OsWRKY7‐diORF‐OE*) were generated by transformation of the *35S::OsWRKY7(−A)‐FLAG* construct. Nine independent transgenic lines were subjected to *Xoo* infection, and significant decreases in length of lesions were found in the three T_1_ lines with high protein levels (Figure 5a,b). Consistent with the enhanced *Xoo* resistance, the transcript levels of the PR genes *PR1a*, *PR1b*, *PR5*, and *PR10a* were up‐regulated in these overexpressing plants and the levels remained high or were greatly increased after *Xoo* infection (Figure 5c). These results suggest that the N‐terminal‐truncated OsWRKY7 protein from alternative translation positively regulates rice innate immunity to *Xoo*.

We also noticed that hypersensitive response (HR)‐specific brown lesions appeared on leaves of the *OsWRKY7‐diORF‐OE* plants when infected by *Xoo* at the seedling stage (Figure 5d, upper panels). We next determined the H_2_O_2_ levels after *Xoo* infection by 3,3‐diaminobenzidine (DAB) staining. In WT leaves, DAB staining was weak showing that H_2_O_2_ accumulation was low after pathogen infection (Figure 5d, lower panels), whereas in the leaves of *OsWRKY7‐diORF‐OE* plants, dark DAB staining colocalized with the necrotic lesions (Figure 5d, lower panels), implying the accumulation of a large amount of H_2_O_2_. Interestingly, the H_2_O_2_ level was also increased in the mock inoculated leaves (Figure S8a), but the induction pattern was different to that of *Xoo* infection, probably induced by wounding as the intact leaves had no staining (Figure S8b). Accordingly, ROS‐producing genes like respiratory burst oxidase homologue gene *OsRbohB* and *OsRbohE* were found up‐regulated in the OE plants (Figure 5c). These results suggest that overexpression of the short stable OsWRKY7 protein activates the production of ROS and ROS‐mediated cell death.

Since high constitutive expression of the full‐length OsWRKY7 is destructive to plant growth, we assessed the growth of the *OsWRKY7‐diORF‐OE* plants. The overall growth of *diORF‐OE* plants was similar to that of the WT in terms of flowering time and panicle number (Figure S9a). However, there were decreases in most of the agronomic traits measured and the grain number per panicle was more significantly lower (Figure S9b).

### Translation disruption of the mORF of *OsWRKY7* enhances the diORF translation and the resistance to bacterial blight

As we known that uORF often suppresses the translation of the mORF, it is not clear whether the diORF translation is also overwhelmed by the mORF. To answer this question, we replaced the diORF of *OsWRKY7* with Luc gene to construct the *N84‐Luc* vector, and disrupted the first ATG of *N84‐Luc* by removing A (*N84(−A)‐Luc*) (Figure 6a). Both the vectors were expressed under the native promoter of *OsWRKY7* in protoplasts. As shown in Figure 6b, the relative Luc activity of *N84(−A)‐Luc* was higher than *N84‐Luc*, suggesting the Luc protein level was increased after preventing the translation from the main AUG. Then, we generated transgenic plants expressing the Flag tag‐fused *OsWRKY7* and the first AUG mutant under the native promoter, and compared the protein levels between lines with the most strongest signals in each transgenic population (Figures S10a,b). Similarly, the short protein levels in the *pOsWRKY7::OsW7(−A)‐FLAG* lines were much higher than both proteins in the *pOsWRKY7::OsW7‐FLAG* lines (Figure 6c). These data indicated that stopping translation of the full‐length OsWRKY7 protein resulted in higher protein level of the short form caused by increased translation from the diORF. As a result, the disease resistance in *pOsWRKY7::OsW7(−A)‐FLAG* lines were increased (Figure S10c,d).

Based on the result that the short OsWRKY7 protein could be translated efficiently from the diORF in the absence of the main AUG codon under the native promoter (Figure 6b,c), it appeared probable that the functional short stable isoform could be overexpressed by eliminating the main ATG site via CRISPR/Cas9. After analysing the genomic sequence of *OsWRKY7*, a PAM site (CGG) was found in an optimal position 4 nt downstream from the main ATG (Figure 6d), that would be expected to lead to Cas9 cleavage between A and T. Then, the sequence upstream of the PAM site was selected as sgRNAc for transformation (Figure 6d). After sequencing analysis (Figure S11a), two different nucleotide mutation types were identified, and both had an incomplete ATG with A deletion (Figure 6d).

To test for bacterial blight resistance, two homozygous lines (*oswrky7‐Cas9‐c*) were inoculated with *Xoo*. At 14 dpi, both lines exhibited enhanced resistance with much shorter leaf lesion lengths than Nip WT plants (Figure 6e,f). The remaining lines were all tested for resistance to *Xoo* strain PXO341. Significantly shorter lesion lengths were measured on leaves of the mutant lines, but not on those of the Nip or lines without mutation (Figure S11b,c). The transcripts of the PR genes and *OsRboh* genes were elevated in the mutant plants and were induced in response to *Xoo* infection (Figure 6g). In addition, *oswrky7‐Cas9‐c* mutant plants were strongly resistant to the highly virulent *Xoo* strain PXO99 (Figure S12a), while the *oswrky7‐Cas9‐a* mutant plants were susceptible (Figure S12b), suggesting that *OsWRKY7* may mediate a broad‐spectrum resistance to rice bacterial blight. In accordance with the resistant phenotype, HR‐specific brown lesions appeared on leaves of *oswrky7‐Cas9‐c*‐mutant plants upon *Xoo* infection at either the seedling or the booting stage (Figure 6i, upper panels; Figure S11d), and a high level of H_2_O_2_ was detected in the infected area with necrotic lesions by DAB staining (Figure 6i, lower panels), whereas mock infection with H_2_O did not induce lesion formation or H_2_O_2_ accumulation (Figure S13). Taken together, these results suggest that multiple signalling pathways involving defence gene expression and ROS production are activated in the mutant plants when the main ATG of *OsWRKY7* was impaired by CRISPR/Cas9. Interestingly, mild growth trade‐offs were observed in these plants grown in normal condition (Figure S14). For practical application, we eliminated the main ATG of *OsWRKY7* allele in an elite rice *japonica* cultivar ZJ70 by the sgRNAc target. Likewise, the mutant lines were much more resistant to *Xoo* infection than the ZJ70 wild type in the patch field (Figure 6i).

### The significance of alternative translation for other *OsWRKY* genes phylogenetically related to *OsWRKY7*

Since alternative translation has rarely been reported in plants, we questioned whether the case of *OsWRKY7* is unique among the rice *WRKY* gene family. After a literature search, we found that overexpression of *OsWRKY67‐Myc* in transgenic plants driven by the 35S promoter produced two fusion bands, which were retained after lambda phosphatase treatment (Vo *et al*., 2018). Phylogenetic analysis of the rice *WRKY* gene family revealed that *OsWRKY67* clusters closely with *OsWRKY7* in the same group (II) (Xie *et al*., 2005), and here we show that *OsWRKY67* and two other closely related homologues *OsWRKY10* and *OsWRKY26* also have dual initiation from both the main and diAUG when driven by the 35S promoter (Figure S15a). Disruption of the main AUG by removing ‘A’ led to the expression of the second diORF of *OsWRKY10* and *OsWRKY67* or the third diORF of *OsWRKY26* (Figure S15c). Interestingly, like OsWRKY7, the full‐length proteins of OsWRKY10 and OsWRKY26 were both unstable and accumulated after MG132 treatment (Figure S15a), while the proteins translated from their diORFs were consistently abundant whether treated with MG132 or not (Figure S15a,c), suggesting the existence of a degradation domain at the N‐terminal region, although their N‐terminal amino acid sequences are not well conserved (Figure S16). On the other hand, *WRKY* genes like *OsWRKY3*, *OsWRKY5*, and *OsWRKY14* which are phylogenetically distant from *OsWRKY7* have normal translation controlled by the same 35S promoter, even though they all contain diORFs (Figure S15b). These results demonstrate the significance of coding sequence context for alternative translation.

## Discussion

### OsWRKY7 is a positive regulator in rice basal defence against bacterial blight but a negative regulator on growth

An important step towards the understanding the regulation of the plant defence system is to identify transcriptional regulators responsive to pathogen attack (Liu *et al*., 2005). Through a comprehensive expression analysis of the *WRKY* gene superfamily in rice infected by pathogens, 12 genes were found differentially regulated by an incompatible bacterial blight pathogen (Ryu *et al*., 2006). Among these genes, *OsWRKY11*, *OsWRKY30*, *OsWRKY67*, and *OsWRKY10* have been reported to play positive roles in basal or *Xa*‐gene‐mediated resistance in rice (Choi *et al*., 2020; Han *et al*., 2013; Lee *et al*., 2018; Vo *et al*., 2018). Here, we show that *OsWRKY7* is another important regulator in establishing the basal resistance to bacterial blight through both transcriptional and post‐translational regulation. In the compatible japonica cultivar *Nipponbare*, the *OsWRKY7* transcript levels increased after inoculation with *Xoo* (Figure 1a), but there was no induction in a highly susceptible cultivar IR24 (Figure 1b), knockout of *OsWRKY7* in the Nip background increased susceptibility to both PXO341 and PXO99 (Figure 1e,f; Figure S13b) and impaired the activation of *PR* genes (Figure 1g), suggesting the existence of a basal defence response in *Nipponbare* mediated by *OsWRKY7*. Interestingly, two OsWRKY7 proteins were produced by alternative translation with different stabilities (Figure 2). Plants overexpressing the unstable full‐length OsWRKY7 protein enhanced disease resistance but inhibited plant growth (Figure 3d–f). In fact, it is not easy to obtain transgenic plants overexpressing the full‐length OsWRKY7 under the *Ubi* promoter, which suggested that the high level of full‐length protein may have a detrimental effect on growth and developmental processes. Increasing the level of the alternative protein also elevated the resistance to *Xoo*; however, obvious growth inhibition was observed when controlled by 35S promoter (Figure S9). These results revealed the importance of the OsWRKY7 protein homeostasis in balancing growth and defence.

### Differential usage of two in‐frame translational start codons regulates OsWRKY7 protein stability

Generally, translation initiation in eukaryotes is based on the ‘first‐AUG rule’ (Kozak, 1987, 2002), but sometimes this rule is abrogated, and different proteins can be produced from a single transcript, for example, by dual initiation at both the first and downstream AUG codons (Slusher *et al*., 1991; Song *et al*., 2009). Mechanisms accounting for this escape have been elucidated extensively at the molecular level (Gray and Wickens, 1998; Kozak, 1994), but the biological functions behind these mechanisms of regulation are still largely unknown. In *Arabidopsis*, it has been reported that targeting of THI1 protein to both mitochondria and chloroplasts is regulated by the alternative use of two in‐frame AUG codons (Chabregas *et al*., 2003). Here, we show that two protein isoforms of OsWRKY7 translated from two in‐frame AUG codons are similar in subcellular localization but differ in protein stability (Figures 3 and 4; Figure S7). In the study of a human opioid receptor OPRM1, a short‐lived isoform was generated by initiation at an alternative in‐frame upstream AUG site (uAUG) in the 5′‐untranslated region, which was subsequently degraded by the ubiquitin–proteasome pathway through the lysine residues within the extended N‐terminus (Song *et al*., 2009). Although we showed that OsWRKY7 was ubiquitinated (Figure 3c; Figure S6), there are no lysine residues in the N‐terminal degradation domain, and we therefore hypothesize that OsWRKY7 may undergo lysine‐independent ubiquitination (McClellan *et al*., 2019). It will be interesting to determine the mechanism for selective degradation of OsWRKY7 proteins from alternative translation.

### Degradation of OsWRKY7 protein is dependent on the proteasome‐mediated pathway

Proteasome‐mediated degradation of defence proteins is essential for optimal plant growth and development by preventing the untimely activation of defence responses under normal conditions. Many immune regulators are targets of the ubiquitin–proteasome system, but there is limited information about proteasome‐mediated degradation of the WRKY transcription factors despite the large size of this gene family. It has been reported that OsWRKY45, one of the central regulators of the SA/BTH‐induced defence signalling pathway in rice, is regulated by UPS‐dependent degradation (Matsushita *et al*., 2013). Other WRKY proteins like OsWRKY6 and OsWRKY11 are also possibly degraded through the ubiquitin/26S proteasome pathway (Choi *et al*., 2015; Lee *et al*., 2018). In this study, we demonstrate that OsWRKY7 protein stability is also controlled by the ubiquitination‐mediated proteasome pathway (Figure 3c; Figure S6). However, the mode of degradation may differ between these proteins. For example, the degradation domain of OsWRKY7 is located at the N‐terminal region, and is separate from both the nearby activation domain and the C‐terminal WRKY DNA‐binding domain, whereas, in OsWRKY45, the domains required for degradation and transcriptional activity closely overlap in the C‐terminal region, and as a result, deletion of the degradation domain also compromises its strong blast resistance (Matsushita *et al*., 2013). In contrast, the truncated OsWRKY7 protein without the degradation domain had increased disease resistance to bacterial blight (Figure 5a,b). In addition, the nuclear localization of OsWRKY45 was necessary for its degradation (Matsushita *et al*., 2013), but the degradation of OsWRKY7 can occur outside of the nuclei as demonstrated by the cytoplasmic localization of the unstable CT1 fragment (Figure 4d).

### The possible mechanism for alternative translation of *OsWRKY7* and closely related genes

In eukaryotes, two independently initiated proteins from one mRNA are generally produced by context‐dependent leaky scanning (Kozak, 1991, 1994; Lin *et al*., 1993). The optimal sequence for translation initiation in vertebrates is GCC*R*CCAUGG (R at −3, is A or G; the AUG initiation codon is underlined) and is known as the Kozak motif (Kozak, 1986). Positions ‐3R (most often A) and +4G are the most conserved and crucial nucleotides (Kozak, 1981, 1984, 1986). Although the Kozak motif varies among different eukaryotes, the ‐3R and +4G are conserved in species of green plants (Gupta *et al*., 2016; Hernandez *et al*., 2019; Rangan *et al*., 2008) and confer the best translational efficiency tested experimentally in many plant species including *Oryza sativa* (Sugio *et al*., 2010). We therefore analysed the native sequence context flanking the AUG initiation codon in *OsWRKY7* and the related *WRKY* genes tested in this study (Table S4). These sequences were categorized based on the presence of the two crucial nucleotides at −3 and +4 in their Kozak motifs (Meijer and Thomas, 2002). It is interesting to find that most of the *WRKY* genes have strong (both of the key nucleotides are present) or adequate (only one of the key nucleotides is present) Kozak motifs at their native initiation site and only *OsWRKY26* has a weak Kozak motif (lacking both key nucleotides). So the alternative translation observed in this study cannot be fully explained by context‐dependent leaky scanning. Other sequence features such as the 5′‐untranslated leading sequence and downstream secondary structure may influence the processes of translation initiation (Kozak, 1990, 1991, 1994). Indeed, we found that the full‐length *OsWRKY7* sequence under control of the *Ubi* promoter did not produce two isoforms (Figures 2a and 3b). The *Ubi* promoter contains 899 bp of the promoter sequence, 83 bp of 5′ untranslated exon, and 1010 bp of first intron sequence from the maize ubiquitin (*Ubi‐1*) gene. (Christensen and Quail, 1996). In most cases, 5′‐untranslated region (5′‐UTR) that enable efficient translation are short, have a low GC content, are relatively unstructured and do not contain uAUG codons (Kochetov *et al*., 1998). 5′‐UTR in the *Ubi* promoter may well consistent with these features for stringent translation. Many studies also showed that intron present in the 5′‐UTR strongly enhanced transgene expression (Chung *et al*., 2006; McElroy *et al*., 1990) by multiple mechanisms including translational control (Laxa, 2017; Rose, 2019). Besides the effect of the promoter, we found that not all *WRKY* genes tested would produce two protein isoforms under the same 35S promoter (Figure S15b), suggesting that the features on the coding sequence may also affect the translation initiation. Further studies are necessary to determine the underlying mechanism for the dual translation initiation of *OsWRKY7* and other closely related genes.

### A proposed working model for OsWRKY7 in the activation of basal defence against bacterial blight

**Figure 7:**
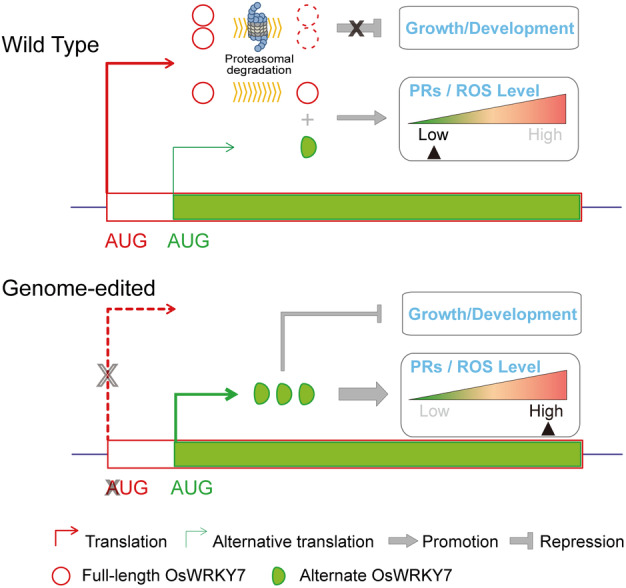
Working model of OsWRKY7 alternative translation during plant basal defence activation in rice. In wild‐type plants, the OsWRKY7 protein translation can be initiated at either the first or the second in‐frame AUG. Under normal condition, the full‐length OsWRKY7 proteins are degraded by the ubiquitin–proteasome system to minimize its inhibition of plant growth and development. On the other hand, the alternate stable OsWRKY7 protein is translated with less efficiency to maintain a low level of basal defence together with the undegraded full‐length protein. In the genome‐edited plants where the first AUG of *OsWRKY7* is disrupted, the alternate OsWRKY7 proteins are accumulated due to the enhanced translation at the second AUG; thus, the basal defence is elevated to a high level through the enhanced defence‐related gene expression and ROS production, while the plant growth and development is partially inhibited.

When plants are subjected to pathogen attack, it would be an efficient coping strategy to regulate defence responses both at the transcriptional level and at the protein level. We find that *OsWRKY7* is such a kind of disease resistance gene with multiple layers of regulation. In addition to its transcriptional induction by the bacterial pathogen *Xoo* (Figure 1a), the translated OsWRKY7 protein is degraded by UPS‐mediated pathway (Figure 3; Figure S6) but induced by Flg22 and *Xoo* pathogen treatment (Figure 3g,h). In addition, the stability of OsWRKY7 is also modulated by alternative translation, which produced a functional N‐terminal truncated isoform that resists UPS‐mediated degradation (Figures 2 and S7). Here, we propose a model of *OsWRKY7* in the regulation of basal defence responses (Figure 7). In uninfected rice plants, the full‐length OsWRKY7 proteins are degraded by the UPS system to minimize negative effect on plant growth. Meanwhile, short stable OsWRKY7 isoforms are generated by alternative translation with less efficiency to provide a constant basal level of defence. In the genome‐edited plants where the main AUG is disrupted, removal of the suppression on the diORF translation enhances the endogenous expression of the short stable OsWRKY7 protein, thus the plant basal defence against bacterial blight is promoted by increased defence‐related gene expression and ROS accumulation, along with a partial inhibition on growth. This study provides insights into the mechanisms of alternative translation and protein turnover in the regulation of OsWRKY7‐mediated basal defence in plants, and also provides a practical strategy to breed disease‐resistant rice by translational regulation of the *OsWRKY7* alleles via genome editing at the main ATG.

## Experimental procedures

### Plant materials and growth conditions

Rice (*Oryza sativa*) plants used to determine the expression of *OsWRKY7* were the *japonica* variety *Nipponbare* (Nip) and *indica* variety IR24. Transgenic overexpression and genome‐edited plants of *OsWRKY7* were generated in the *Nipponbare* or ZJ70. Rice seeds were germinated in petri dishes with water at 37 °C and hydroponically cultured in the rice nutrient solution (Yoshida *et al*., 1976) in a growth chamber with a 14‐h light (30 °C)/10‐h dark (28 °C) photoperiod. *Indica* variety 9311 was used for transient gene expression in protoplasts and cultured on 1/2 MS medium for 7–10 days in the same growth chamber.

### Vector construction and plant transformation

The detailed vector construction information was listed in supporting information of method. *Agrobacterium*‐mediated transformation was conducted as previously described (Hiei and Komari, 2008).

### Bacterial blight inoculation

Two *Xanthomonas oryzae* pv. *oryzae* (*Xoo*) races from the Philippines (PXO341 and PXO99) were used. Strains were cultured in a in modified Wakimoto's medium at 28 °C to the optical density of OD_600_ = 0.6–0.8. Fully expanded rice leaves were clipped about 1–2 cm from the tip by scissors dipped with bacterial suspension or sterile deionized water (Kauffman *et al*., 1973). To analyse *OsWRKY7* gene expression in response to *Xoo*, leaves of 3‐week‐old *Nipponbare* and IR24 were inoculated with PXO341 or H_2_O for 12, 36, and 60 h, and 2 cm leaf tissues below the cutting edge were collected at the indicated time points. To analyse *PR* gene expression, 3‐week‐old seedlings were inoculated with PXO341 or H_2_O for 48 h. For evaluation of bacterial blight resistance, plants at the booting stage (70 days after sowing) were inoculated with PXO341 or PXO99, the lesion length was measured 2 weeks after inoculation. To analyse protein levels upon *Xoo* treatment, Plantlets were sprayed with PXO341 suspension containing 0.05% [v/v] Silwet L‐77 for 6 h. H_2_O + Silwet L‐77 used as mock control.

### Recombinant protein purification and cell‐free degradation assay

GST‐OsWRKY7 recombinant protein was induced in *E. coli* BL21(DE3) and purified by glutathione affinity resin column (Pierce，16100). The cell‐free degradation assays were performed as previously described (Wang *et al*., 2009). Briefly, total proteins from 100 mg WT leaves were extracted in 1 mL degradation buffer. Then, the supernatants were collected by centrifugation at 13 000 rpm for 10 min at 4 °C. A total quantity of 100 ng purified GST and GST‐OsWRKY7 were incubated in 100 μL protein extracts without (−) or with (+) 100 μM MG132 at 28 °C for 0, 0.5, 1, 2, and 3 h. The protein abundance was detected by an anti‐GST antibody (1:10 000, Abmart, M20007), followed by a secondary antibody (1:5000, Abbkine, A21010). Coomassie blue‐stained Rubisco large protein (RubL) was used as a loading control.

### Transient gene expression in rice protoplasts

Rice protoplasts preparation and transformation were conducted according to the method of Zhang *et al*., 2011. The sheaths and stems of 30–40 seedlings were cut into 0.5 mm strips and incubated immediately in 10 mL enzyme solution for 4–5 h in the dark at 25 °C with gentle shaking (60 rpm). The protoplasts were purified and resuspended in 1–2 mL MMG solution at a concentration of 5 × 10^6^ cells mL^−1^. Plasmids (5–10 μg) prepared by an EndoFree Plasmid Midi Kit (CWBIO, Beijing, China) were used for transfection. For the protein degradation assay, DMSO (mock) or 20 μM MG132 (Millipore), H_2_O (0 μM), 1, 5, and 25 μM E‐64 (Sigma‐Aldrich) or Leupeptin (Sigma‐Aldrich) were added to the protoplasts 12 or 4 h after transfection and treated for 4 or 12 h. For the time course treatment, 20 μM MG132 and/or 50 μM cycloheximide (CHX, Sigma‐Aldrich) were added to the protoplasts 12 h after transfection and treated for 2 h, 4 h, and 6 h. For pathogen mimic treatment, H_2_O (0 μM), 0.5, 1, 2, and 5 μM Flg22 (Sangon Biotech) were added to the protoplasts 16 h after transfection and treated for 2 h. Transfection experiments were repeated at least three times.

### In vivo ubiquitination assay

*Ubi::OsWRKY7‐3 × FLAG* and/or *35S::Myc‐Ubi* were transiently expressed in rice protoplasts and treated with or without MG132 (50 μM). Proteins were extracted from protoplasts in 500 μL IP lysis buffer (Pierce, Thermo Scientific) plus 1 × plant protease inhibitor cocktail (Thermo Scientific). After centrifugation at 100 ***g*** for 10 min at 4 °C, the supernatant was mixed with 5 μL anti‐FLAG magnetic beads (Genscript, L00790‐1) and incubated at 4 °C for 2 h. After washing, the IP products were boiled at 100 °C for 10 min. Samples were separated by 10% Bis‐Tris PAGE Gel (GenScript, Nanjing, China) and subjected to immunoblot analysis. The ubiquitin modifications were detected with the anti‐Myc (1:10 000, Abmart, M20002) and anti‐Ubi P4D1 (1:1000, Santa Cruz, sc‐8017) antibodies. The immunoprecipitated OsWRKY proteins were detected with the anti‐FLAG (1:10 000, GenScript, A00187) antibody.

### Luciferase transactivation assays

Seven micrograms of *OsPR10a::LUC* reporter plasmid generated previously (Ersong *et al*., 2021) was transfected with 3 μg effector plasmid. Five micrograms of *0800‐Luc*, *N84‐Luc*, and *N84(−A)‐Luc* vectors were transfected alone. Firefly luciferase (LUC) and renilla luciferase (REN) activities were measured using the Dual‐Luciferase Reporter Assay System (Promega). The luminescence signals were detected using the microplate reader SpectraMaxi3. Relative LUC activity was calculated by normalizing the value of LUC to REN in each sample. Triple transfections were carried out for each reporter/effector combination in one experiment, and two experiments were performed giving comparable results.

### DAB staining

H_2_O_2_ accumulation was detected using the 3,3′‐diaminobenzidine (DAB; BBI, Shanghai, China) uptake method as described previously with modification (Thordal‐Christensen *et al*., 1997). The second leaves from top of 25‐day‐old seedlings were cut 5 days after inoculation with PXO341 or H_2_O_2_, and immediately submerged in DAB solution (1 mg mL^−1^ DAB in 0.1 M Tris–HCl, and pH = 3.8) at 25 °C for 10 h in the light. Leaves were then de‐stained in bleaching solution (acetic acid:ethanol = 1:1) at 37 °C for 12 h until all of the chlorophyll had been removed. The decolorized leaves were photographed under a Nikon SMZ1000 stereomicroscope equipped with a Nikon digital camera DS‐Fi1.

### Statistical analysis

Statistical analysis was performed by two‐tailed Student's *t*‐test and one‐way ANOVA with Tukey's or Newman–Keuls multiple comparison test.

## Conflicts of interest

The authors declare no conflicts of interest.

## Author contributions

J.Z. and X.W. designed the experiments; C.Z., J.Z., and X.Y. performed the experiments with assistance from E.Z., X.L., W.C., C.Y., and Y.W.; J.Z., W.R., and K.Y. analysed the results; J.Z., X.W., and J.C. wrote the article.

## Supporting information


**Figure S1** Characterization of the *OsWRKY7* loss of function mutant rice plants generated by CRISPR/Cas9‐mediated mutagenesis.
**Figure S2** Generation of the *OsWRKY7* loss of function rice plants by CRISPR/Cas9‐mediated mutagenesis at the sgRNAb site.
**Figure S3** Alternative splicing analysis of OsWRKY7 gene transcription from RNA‐seq data of *Nipponbare*.
**Figure S4** LC–MS/MS analysis of the proteins translated from the *OsWRKY7‐SR* gene under control of the 35S promoter.
**Figure S5** OsWRKY7 protein was not degraded through the lysosomal pathway.
**Figure S6** In vivo ubiquitination assay of OsWRKY7 protein.
**Figure S7** MG132 and CHX time course treatment of the full‐length and short OsWRKY7 proteins.
**Figure S8** H_2_O_2_ levels in leaves of WT and *OsWRKY7‐diORF‐OE* transgenic plants without *Xoo* infection.
**Figure S19** The agronomic phenotypes of the *OsWRKY7‐diORF‐OE* transgenic plants.
**Figure S10** Characterization of plants transformed with the full‐length and A deletion *OsWRKY7* constructs controlled by the native promoter.
**Figure S11** CRISPR/Cas9 plants with the first ATG of *OsWRKY7* mutated had enhanced resistance to *Xoo* and hypersensitive response (HR)‐related cell death.
**Figure S12** *OsWRKY7* regulated defence response against the highly virulent *Xoo* strain PXO99.
**Figure S13** H_2_O_2_ levels in leaves of WT and *oswrky7‐Cas9‐c* transgenic plants without *Xoo* infection.
**Figure S14** The agronomic phenotypes of the *oswrky7‐Cas9‐c* transgenic plants.
**Figure S15** Alternative translation initiation of *OsWRKY* group II members clustering in the clade with *OsWRKY7*.
**Figure S16** Conservation of OsWRKY7 protein and its closely related homologues.

**Table S1** Sequences of primers used for vector construction.
**Table S2.** Sequences of primers used for RT‐PCR.
**Table S3** LC–MS/MS identified peptides in the proteins expressed from the mutated *OsWRKY7‐SR* gene controlled by 35S promoter.
**Table S4.** Comparison of the AUG initiation codon context (−6 to +4) in the *OsWRKY* genes tested in this study.

**Data S1** Supporting Information.
