# Supplementary material for: Elevating plant immunity by translational regulation of a rice WRKY transcription factor
Source: Plant Biotechnol J. 2023 Nov 23;22(4):1033–48. doi: 10.1111/pbi.14243 (PMC10955491; doi:10.1111/pbi.14243)
Supplement: Supplementary file 1 — Figure S1 Characterization of the OsWRKY7 loss of function mutant rice plants generated by CRISPR/Cas9‐mediated mutagenesis. Figure S2 Generation of the OsWRKY7 loss of function rice plants by CRISPR/Cas9‐mediated mutagenesis at the sgRNAb site. Figure S3 Alternative splicing analysis of OsWRKY7 gene transcription from RNA‐seq data of Nipponbare. Figure S4 LC–MS/MS analysis of the proteins translated from the OsWRKY7‐SR gene under control of the 35S promoter. Figure S5 OsWRKY7 protein was not degraded through the lysosomal pathway. Figure S6 In vivo ubiquitination assay of OsWRKY7 protein. Figure S7 MG132 and CHX time course treatment of the full‐length and short OsWRKY7 proteins. Figure S8 H2O2 levels in leaves of WT and OsWRKY7‐diORF‐OE transgenic plants without Xoo infection. Figure S19 The agronomic phenotypes of the OsWRKY7‐diORF‐OE transgenic plants. Figure S10 Characterization of plants transformed with the full‐length and A deletion OsWRKY7 constructs controlled by the native promoter. Figure S11 CRISPR/Cas9 plants with the first ATG of OsWRKY7 mutated had enhanced resistance to Xoo and hypersensitive response (HR)‐related cell death. Figure S12 OsWRKY7 regulated defence response against the highly virulent Xoo strain PXO99. Figure S13 H2O2 levels in leaves of WT and oswrky7‐Cas9‐c transgenic plants without Xoo infection. Figure S14 The agronomic phenotypes of the oswrky7‐Cas9‐c transgenic plants. Figure S15 Alternative translation initiation of OsWRKY group II members clustering in the clade with OsWRKY7. Figure S16 Conservation of OsWRKY7 protein and its closely related homologues. [file PBI-22-1033-s002.pdf]

(a)

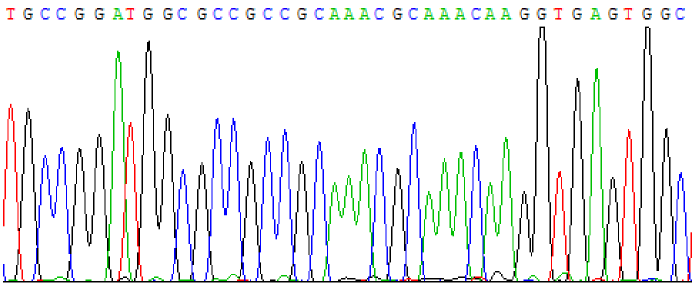

TGCCGGATGGCGCCGCCGCAAACGCAAACAAGGTGAGTGGC Type 1(+1)

TGCCGGATGGCGCCGCCGCAAACGCA ACAAGGTGAGTGGC WT

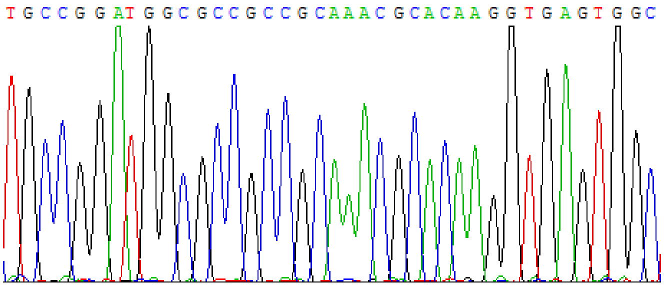

TGCCGGATGGCGCCGCCGCAAACGCACAAGGTGAGTGGC Type 2(-1)

TGCCGGATGGCGCCGCCGCAAACGCAACAAGGTGAGTGGC WT

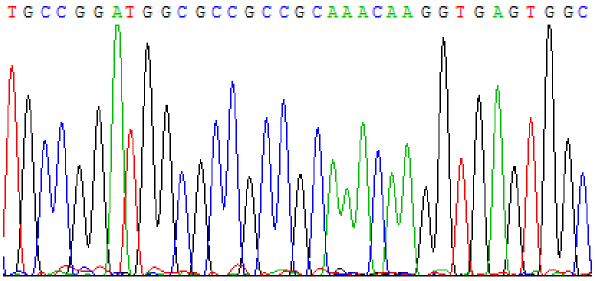

TGCCGGATGGCGCCGCCGCAAACAAGGTGAGTGGC Type 3(-5)

TGCCGGATGGCGCCGCCGCAAACGCAACAAGGTGAGTGGC WT

(b)

```
OsWRKY7-Protein     MAAVGAHAAVYHHPVSGLSAPAGDAAYSMSSYFSHGGSSTSSSASSFSAALAAATTPPLP
Type 1(+1)-No.2     MAAVGAHAAVYHHPVSGLSAPAGDAAYSMSSYFSHGGSSTSSSASSFSAALAAATTPPLP
Type 2(-1)-No.5     MAAVGAHAAVYHHPVSGLSAPAGDAAYSMSSYFSHGGSSTSSSASSFSAALAAATTPPLP
Type 3(-5)-No.6     MAAVGAHAAVYHHPVSGLSAPAGDAAYSMSSYFSHGGSSTSSSASSFSAALAAATTPPLP
                    ************************************************************

OsWRKY7-Protein     DPSGSQFDISEFFFDDAPPAAVFNGAPTAALPDGAAANATRSAAEAVPAPAPAAVERPRT
Type 1(+1)-No.2     DPSGSQFDISEFFFDDAPPAAVFNGAPTAALPDGAAANANKERGGGGAGAGAGG--RGEA
Type 2(-1)-No.5     DPSGSQFDISEFFFDDAPPAAVFNGAPTAALPDGAAANAQGARRRRCRR----------
Type 3(-5)-No.6     DPSGSQFDISEFFFDDAPPAAVFNGAPTAALPDGAAAN--KERGGGGAGAGAGG--RGEA
                    *************************************

OsWRKY7-Protein     ERIAFRTKSEIEILDDGYKWRKYGKKSVKNSPNPRNY-YRCSTEGCNVKKRVERDKDDPS
Type 1(+1)-No.2     ADGADRVPDEVRDRDS--------------------------------------------
Type 2(-1)-No.5     --------------RRRRPWRGRGRSGSRSGRSQRSRFLTTATSGASTARSPSRT--APT
Type 3(-5)-No.6     ADGADRVPDEVRDRDS--------------------------------------------

OsWRKY7-Protein     YVVTTYEGTHNHVSPSTVYYASQDAASGRFFVAGTQPPGSLN
Type 1(+1)-No.2     ------------------------------------------
Type 2(-1)-No.5     QGT--TTGARRKGA--------------------------T-
Type 3(-5)-No.6     ------------------------------------------
```

(c)

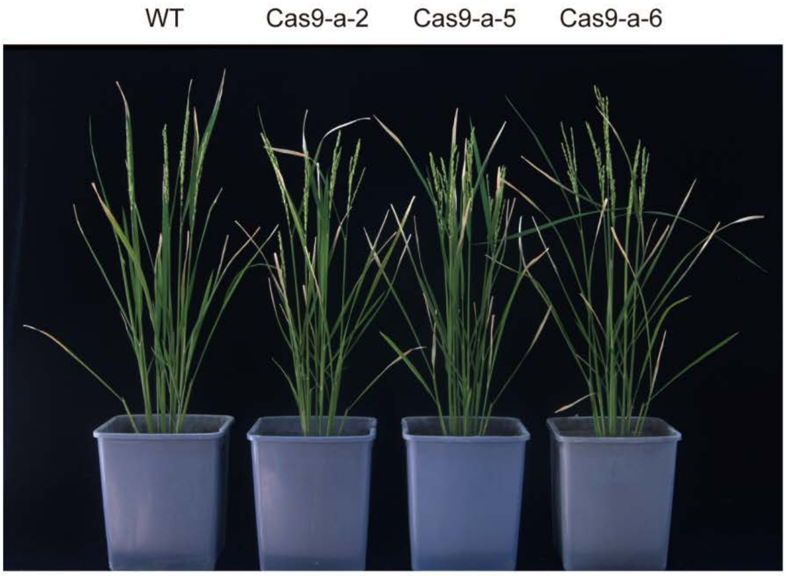

Figure S1 Characterization of the *OsWRKY7* loss of function mutation rice plants generated by CRISPR/Cas9-mediated mutagenesis. (a) Detecting mutations of the *OsWRKY7* sgRNAa-mediated mutagenesis by direct sequencing of PCR products containing the targeted site (highlighted in blue color). The nucleotides were read according to the sequencing chromatogram viewed in Chromas and three types of homozygous mutations are shown. The deleted or inserted nucleotides are highlighted in yellow color on the wild type reference (WT) and the number is given in brackets for each type. The red letters indicate the PAM site. (b) Multiple sequence alignment of the OsWRKY7 proteins decoded from the coding region obtained from the mutant

lines (No. 2, 5, 6) and WT by the Clustal Omega program online (https://www.ebi.ac.uk/Tools/msa/clustalo/). The consensus amino acids are shown by "*". (c) Disease severity in plants of three Cas9-a mutant lines and WT was assessed 15 days after inoculation with *Xoo* isolate PXO341.

(a)

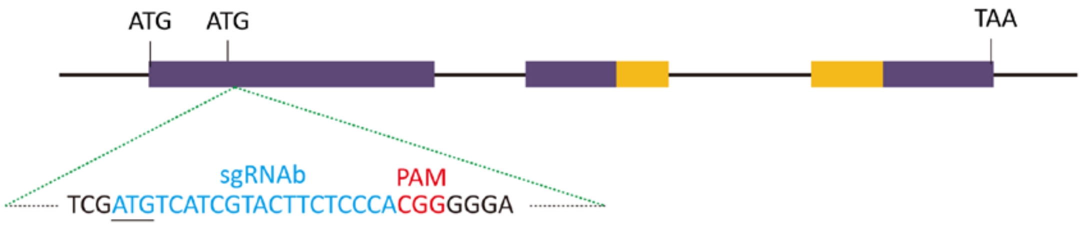

(b)

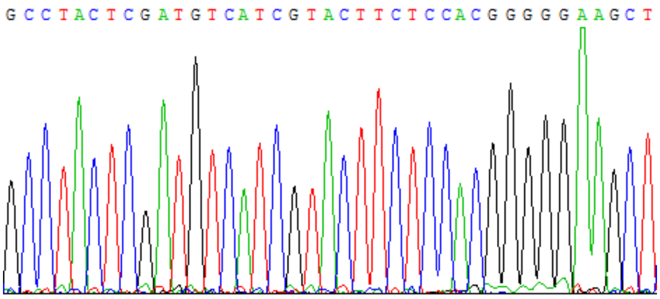

GCCTACTCGATGTCATCGTACTTCTCCACGGGGGAAGCT Type 1(-1)

GCCTACTCGATGTCATCGTACTTCTCCCACGGGGGAAGCT WT

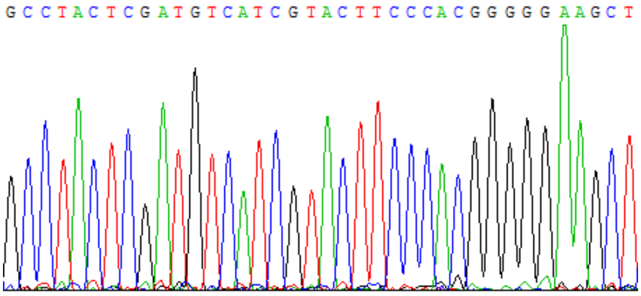

GCCTACTCGATGTCATCGTACTTCCCACGGGGGAAGCT Type 2(-2)

GCCTACTCGATGTCATCGTACTTCTCCCACGGGGGAAGCT WT

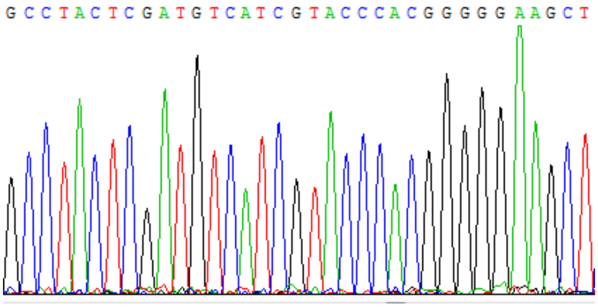

GCCTACTCGATGTCATCGTACCCACGGGGGAAGCT Type 3(-5)

GCCTACTCGATGTCATCGTACTTCTCCCACGGGGGAAGCT WT

(c)

```
OsWRKY7-Protein     MAAVGAHAAVYHHPVSGLSAPAGDAAYSMSSYFSHGGSSTSSSASSFSAALAAATTPPLP
Type 1(-1)          MAAVGAHAAVYHHPVSGLSAPAGDAAYSMSSYFSTGEARPPAPRPAS--------PPPSP
Type 2(-2)          MAAVGAHAAVYHHPVSGLSAPAGDAAYSMSSYFPRGKLDLQLRVQLL--------RR---
Type 3(-5)          MAAVGAHAAVYHHPVSGLSAPAGDAAYSMSS-YPRGKLDLQLRVQLL--------RR---
                     ******************************* :  *

OsWRKY7-Protein     DPSGSQFDISEFFFDDAPPAAVFNGAPTAALPDGAAANATRSAAEAVPAPAPAAVERPRT
Type 1(-1)          PQRRRRFPTR-------------RGRS-STSPSSSSTTRLRPPCSMARPPPRCRMAPPQT
Type 2(-2)          -------PRR-------------RNDAAASRPVGVAVRHLR-------------VLLRRR
Type 3(-5)          -------PRR-------------RNDAAASRPVGVAVRHLR-------------VLLRRR
                                            ..   :: * . :.   *             :   :

OsWRKY7-Protein     ERIAFRTKSEIEILDDGYKWRKYGKKSVKNSPNPRNYYRC---STEGCNVKKRVERDKDD
Type 1(-1)          QQG-A--RRRRCRRRRRRPWRGRGRSGSRSGRSQRSRFL--TTATSGASTARSPSRTAPT
Type 2(-2)          ASG-R--RVQWRAHRRAAGWRRRKRNKERGGGGAGAGAGGRGEAADGAD--RVPDEVRDR
Type 3(-5)          ASG-R--RVQWRAHRRAAGWRRRKRNKERGGGGAGAGAGGRGEAADGAD--RVPDEVRDR
                            : .         **   :.  :.. .          ::.*..  :  ..

OsWRKY7-Protein     PSYVVTTYEGTHNHVSPSTVYYASQDAASGRFFVAGTQPPGSLN
Type 1(-1)          QG----TTTGARR--------------------------KGAT-
Type 2(-2)          DS------------------------------------------
Type 3(-5)          DS------------------------------------------
```

(d)

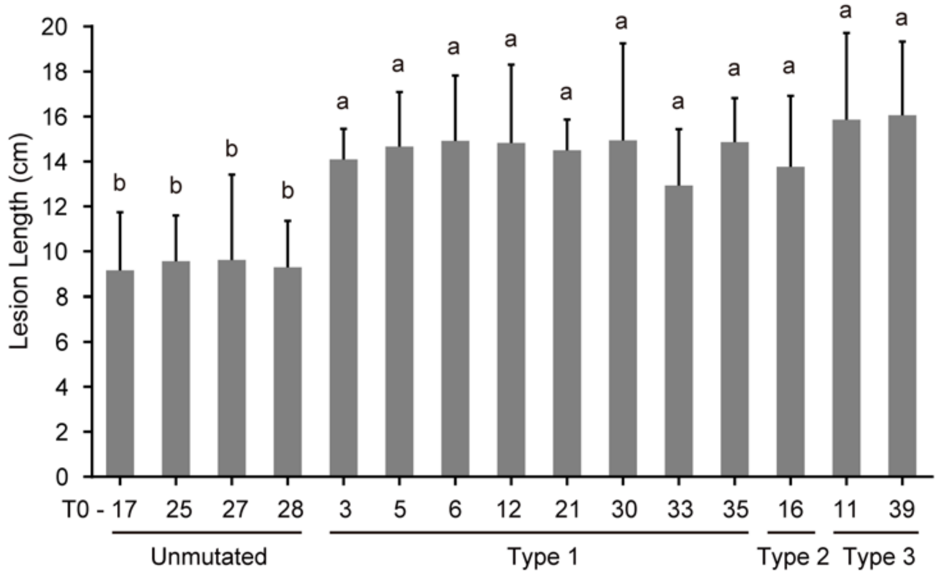

Figure S2 Generation of the *OsWRKY7* loss of function rice plants by CRISPR/Cas9-mediated mutagenesis at the sgRNAb site. (a) The sgRNAb target sequence (blue letters) and the PAM site (red letters) are shown after the second in-frame ATG. (b) Detecting mutations by direct sequencing of PCR products containing the targeted site (highlighted in blue color). The nucleotides were read according to the sequencing chromatogram viewed in Chromas and three types of homozygous mutations are shown. The deleted nucleotides are highlighted in yellow color on the WT reference and the number is given in brackets for each type. The red letters indicate the PAM site. (c) Multiple sequence alignment of the OsWRKY7 proteins decoded from the coding region obtained from the three mutation types at

sgRNAb target in (b) and WT by the Clustal Omega program online (https://www.ebi.ac.uk/Tools/msa/clustalo/). The consensus amino acids are shown by "*". (d) Lesion lengths on leaves of WT and sgRNAb targeted T_0 lines, which had unmutated or mutated target sites shown in (b). Plants were at maximum tillering stage when inoculated with *Xoo* strain PXO341. Bars show mean lesion lengths at 14 dpi±SD (n≥7). Lowercase letters indicate significant differences (one-way ANOVA with Tukey's multiple comparison test, $P<0.05$).

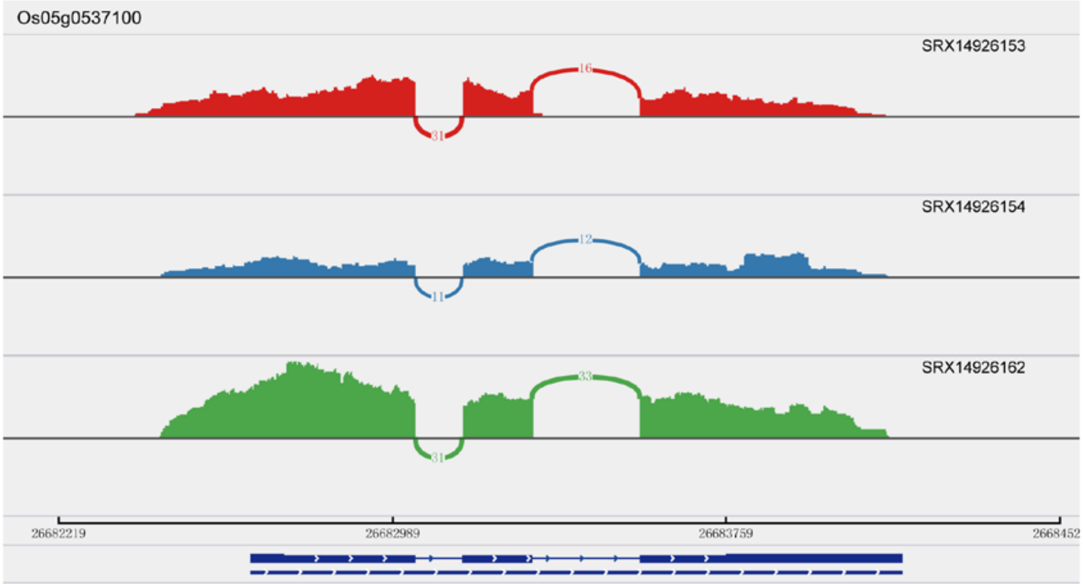

Figure S3 Alternative splicing analysis of *OsWRKY7* gene transcription from RNA-seq data of *Nipponbare*. RNA-seq data of rice cultivar *Nipponbare* were retrieved from the Sequence Read Archive (SRA) in NCBI. Accessions SRX14926153, SRX14926154, and SRX14926162 are three replicate samples of the wildtype *Nipponbare* under normal temperatures for 24 h. The filtered reads were mapped to the reference genome (IRGSP-1.0) using HISAT2 v2.0.5. The rMATS (3.2.5) software was employed to analyze differential variable splicing events of *OsWRKY7* (Os05g0537100). The data is displayed by the Integrative Genomics Viewer (IGV). The number indicates the count of junction reads.

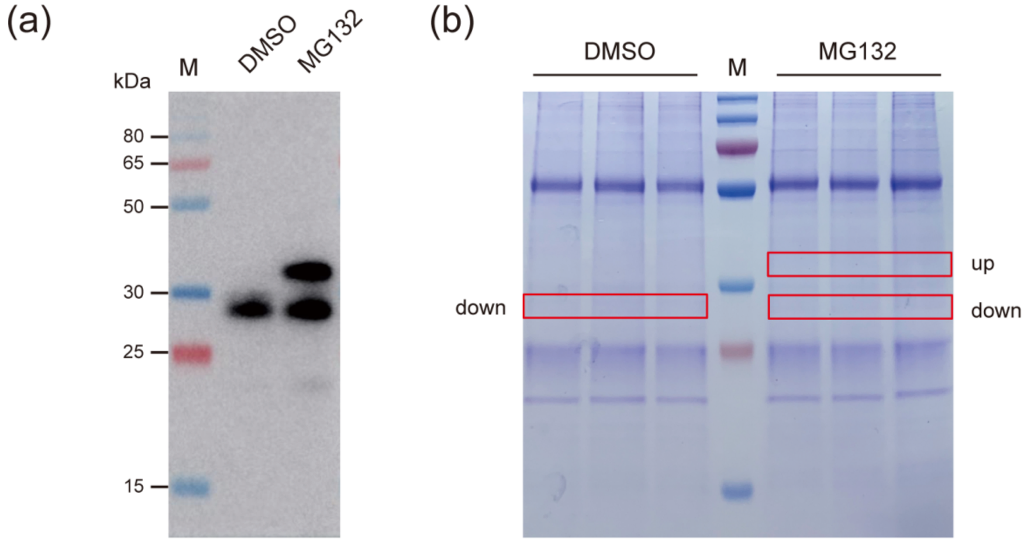

(c)

OsWRKY7-SR-MG132-up

```
  1   MAAVGAHAAV YHHPVRGLSA PAGDAAYRMS SYFSHGGSST SSRASSFSAA
 51   LAAATTPPLP DPSGSQFDIS EFFFDDAPPA AVFNGAPTAA LPDGAAANAT
101   RSAAEAVPAP APAAVERPRT ERIAFRTKSE IEILDDGYKW RKYGKKSVKN
151   SPNPRNYYRC STEGCNVKKR VERDKDDPSY VVTTYEGTHN HVSPSTVYYA
201   SQDAASGRFF VAGTQPPGSL N
```

OsWRKY7-SR-MG132-down

```
  1   MAAVGAHAAV YHHPVRGLSA PAGDAAYRMS SYFSHGGSST SSRASSFSAA
 51   LAAATTPPLP DPSGSQFDIS EFFFDDAPPA AVFNGAPTAA LPDGAAANAT
101   RSAAEAVPAP APAAVERPRT ERIAFRTKSE IEILDDGYKW RKYGKKSVKN
151   SPNPRNYYRC STEGCNVKKR VERDKDDPSY VVTTYEGTHN HVSPSTVYYA
201   SQDAASGRFF VAGTQPPGSL N
```

OsWRKY7-SR-DMSO-down

```
  1   MAAVGAHAAV YHHPVRGLSA PAGDAAYRMS SYFSHGGSST SSRASSFSAA
 51   LAAATTPPLP DPSGSQFDIS EFFFDDAPPA AVFNGAPTAA LPDGAAANAT
101   RSAAEAVPAP APAAVERPRT ERIAFRTKSE IEILDDGYKW RKYGKKSVKN
151   SPNPRNYYRC STEGCNVKKR VERDKDDPSY VVTTYEGTHN HVSPSTVYYA
201   SQDAASGRFF VAGTQPPGSL N
```

Figure S4 LC-MS/MS analysis of the proteins translated from the *OsWRKY7-SR* gene under control of the 35S promoter. (a) Mutated OsWRKY7-SR-3×FLAG protein in which the Ser-16, Ser-28 and Ser-43 were replaced with Arg was transiently expressed in rice protoplasts and treated with 50 μM MG132 or DMSO as mock. Anti-FLAG immunoprecipitated protein was separated in a conventional 15% SDS-PAGE gel and detected with the anti-FLAG antibody. Migration size of the PageRuler Plus prestained protein ladder (M) in MOPS running buffer is indicated. (b)

Coomassie blue staining of the gel after electrophoresis of the remaining immunoprecipitated proteins in the DMSO or MG132 treatments used in (a). Red rectangles indicate the regions of the upper (up) and lower (down) bands that were cut according to the immunoblot size shown in (a) and sent for LC-MS/MS analysis. (c) Alignment of the peptides identified by LC-MS/MS from the gel samples indicated in (b). Peptides matched to the OsWRKY7-SR protein sequence are highlighted in yellow. The first and second Met (M) are shown in bold green and the replaced Arg (R) residues are in bold red.

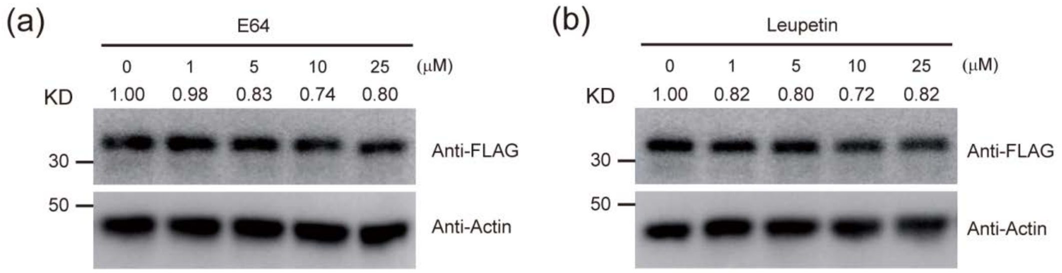

Figure S5 OsWRKY7 protein was not degraded through the lysosomal pathway. (a) The protoplasts transfected with *Ubi::OsWRKY7-3×FLAG* were were treated with 0 μM, 1 μM, 5 μM and 25 μM E-64 (calpain inhibitor) for 12 h. (b) The protoplasts transfected with *Ubi::OsWRKY7-3×FLAG* were treated with 0 μM, 1 μM, 5 μM and 25 μM Leupetin (calpain and trypsin-like inhibitor) for 12 h. Total protein was extracted and detected with anti-FLAG antibody. The level of Actin protein was used as an internal control. The signal intensity of FLAG relative to Actin was calculated for each treatment in (a) and (b), and normalized to the value of the 0 μM treatment which was set to 1.00.

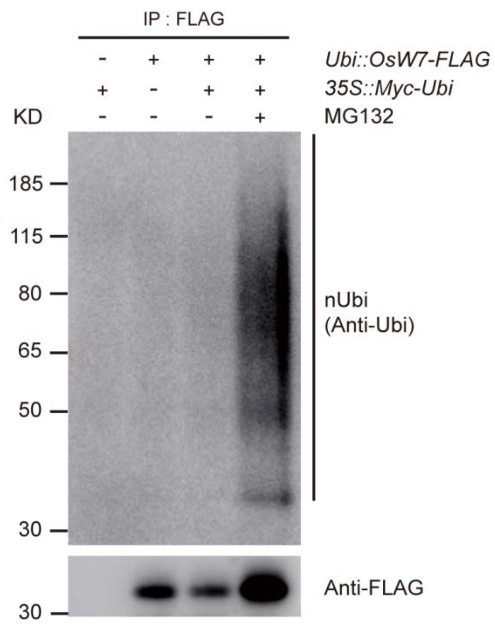

Figure S6 In vivo ubiquitination assay of OsWRKY7 protein. The protein extracts from Figure 3c were tested using anti-Ubi antibody following immunoprecipitation with anti-FLAG magnetic beads. The levels of immunoprecipitated OsWRKY7 proteins were the same as those detected in Figure 3c with anti-FLAG antibody.

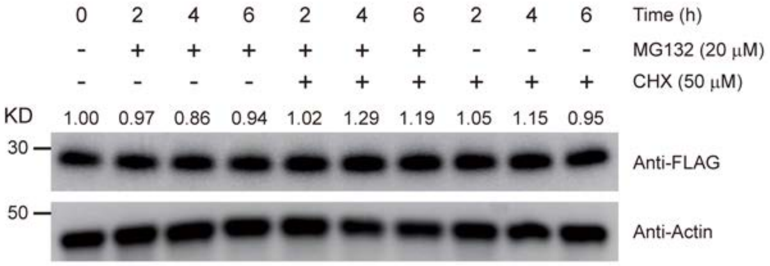

Figure S7 MG132 and CHX time course treatment of the short OsWRKY7 proteins. The protoplasts were transfected with *35S::OsWRKY7(-A)-3×FLAG* to express the short OsWRKY7 protein from the diORF for 12 h and then treated with 20 μM MG132 and/or 50 μM CHX for 2 h, 4 h and 6 h. Total protein was extracted and probed with anti-FLAG and anti-Actin antibodies. The signal intensity of FLAG relative to Actin was calculated for each treatment and normalized to the value of the mock treatment which was set to 1.00.

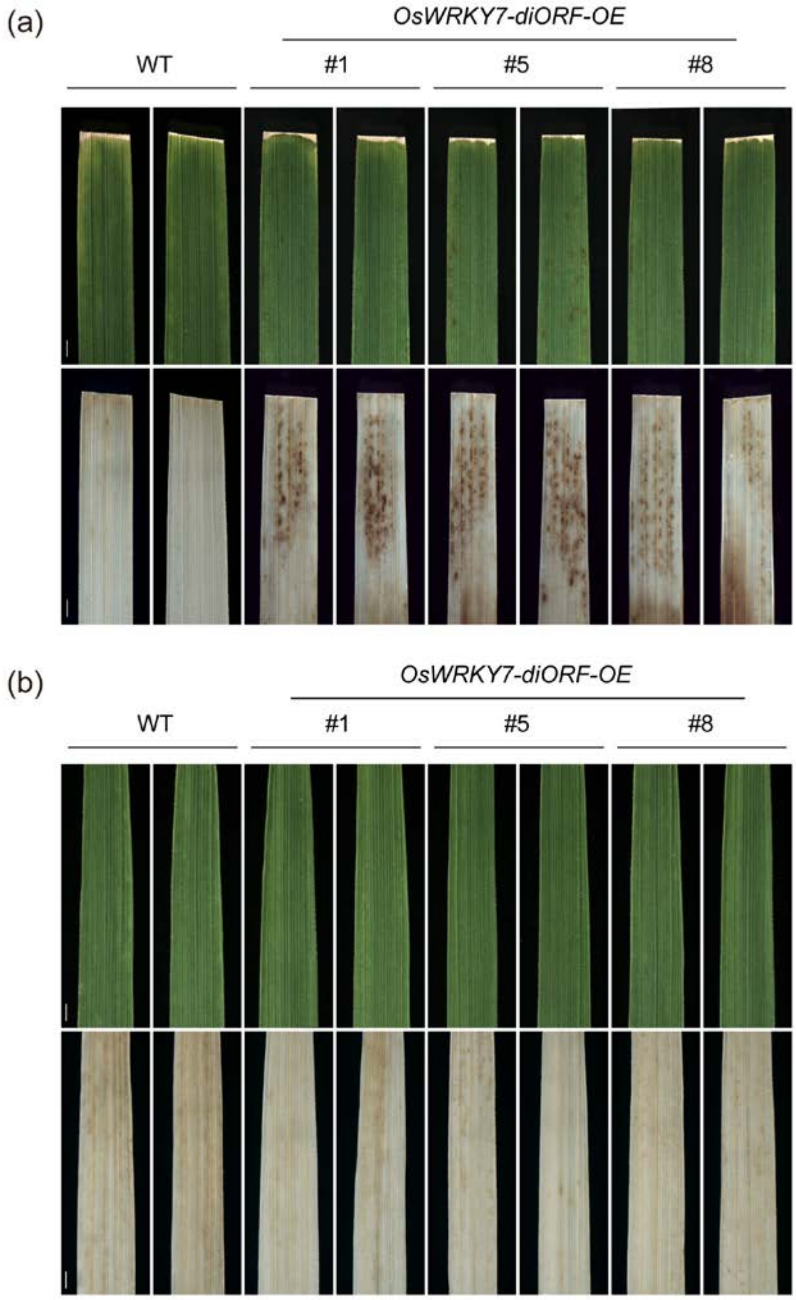

Figure S8 H_2O_2 levels in leaves of WT and *OsWRKY7-diORF-OE* transgenic plants without *Xoo* infection. (a) Leaves mock treated with H_2O at 5 dpi (upper panels). The same leaves were stained with 3,3'-diaminobenzidine (DAB) and photographed after decoloring (lower panels). Scale bars, 1 cm. Two leaves of each line are shown. (b) Intact leaves were photographed (upper panels) and subjected to DAB staining (lower panels). Scale bars, 1 cm. Two leaves of each line are shown.

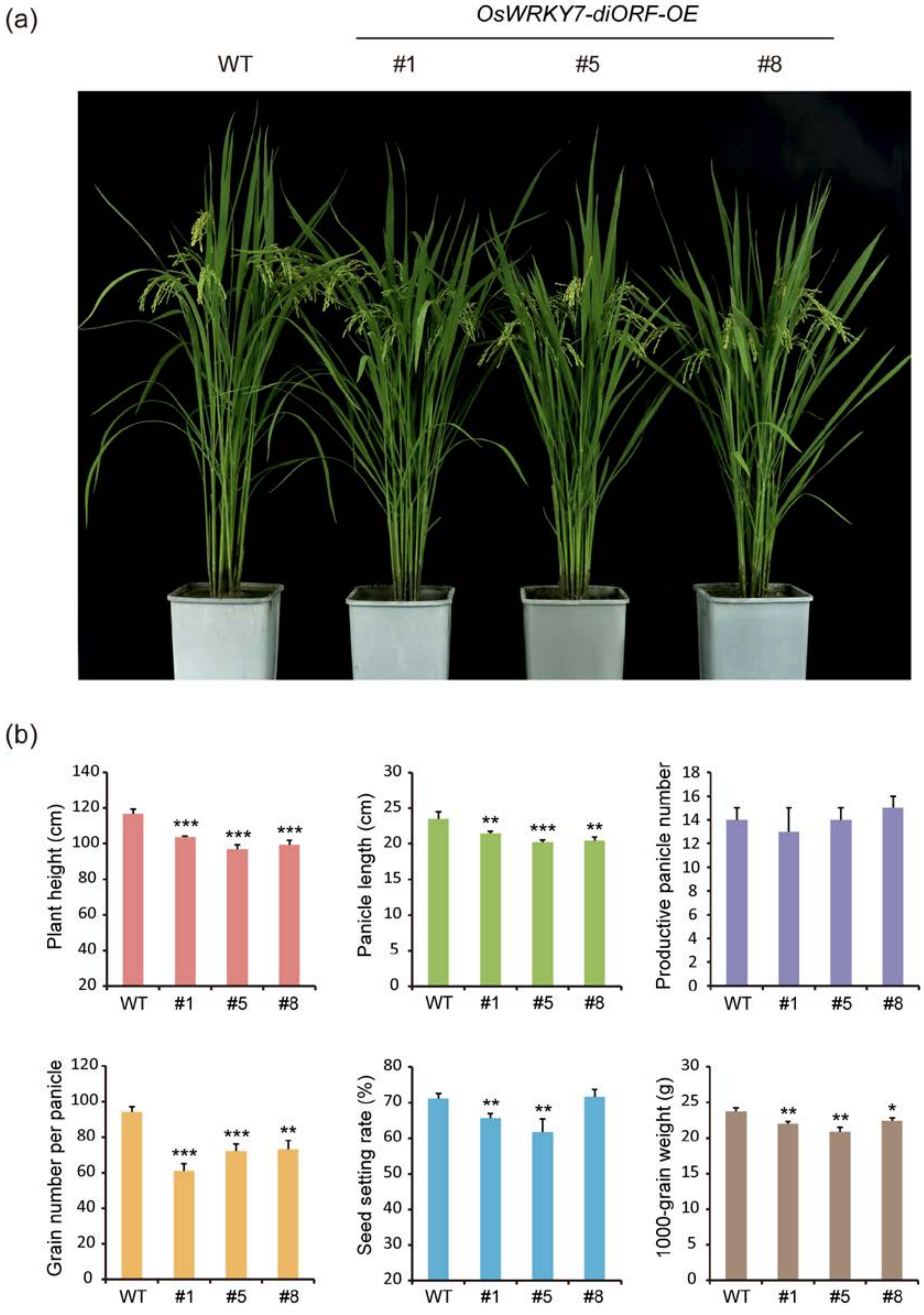

Figure S9 The agronomic phenotypes of the *OsWRKY7-diORF-OE* transgenic plants. (a) Phenotype of transgenic plants overexpressing *OsWRKY7-diORF* at flowering stage. Wild type (Nip) and representative plants from three lines were grown in the same soil plot under natural conditions and photographed at

100-days-old. (b) Agronomic traits in WT and transgenic lines of *OsWRKY7-diORF-OE*, including plant height, panicle length, productive panicle number, grain number per panicle, seed setting rate and 1000-grain weight. The data are means ± SD (n=4). Significant differences between WT and transgenic lines are indicated as *P<0.05, **P<0.01, ***P<0.001 by Student's *t*-test.

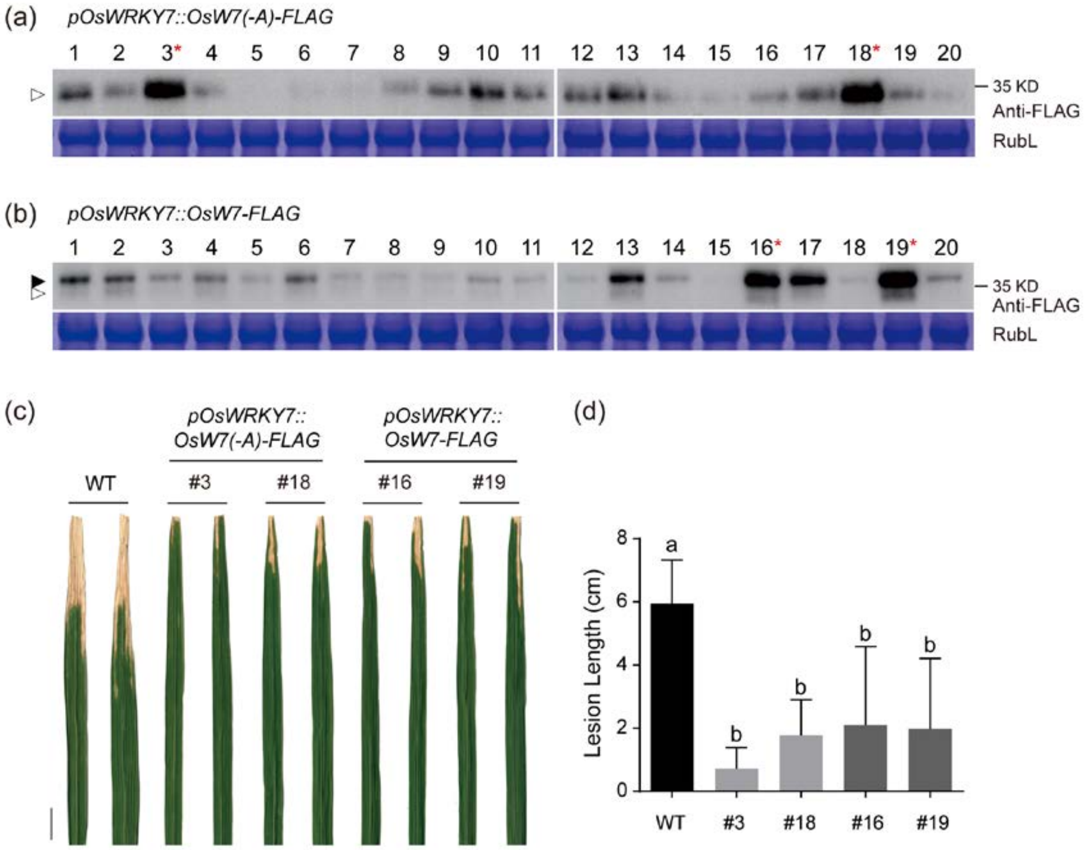

Figure S10 Characterization of plants transformed with the full-length and A deletion *OsWRKY7* constructs controlled by the native promoter. Western blot analysis of the protein levels in transgenic lines expression *OsWRKY7(-A)-FLAG* (a) and *OsWRKY7-FLAG* (b) under *OsWRKY7* promoter. Proteins were detected by anti-FLAG antibody. Lines with strongest signals were selected for comparison. The full length and alternative translated OsWRKY7 proteins were indicated by the black and white arrowheads, respectively. RubL was used as loading control. (c) Lesions on leaves of transgenic lines after PXO341 infection (14 dpi). Scale bar, 2 cm. (d) Lesion lengths at 14 dpi. Bars represent mean lesion lengths ± SD ($n \geqslant 3$). Lowercase letters indicate significant differences by one-way ANOVA with Tukey's multiple comparison test, $P<0.05$.

(a)

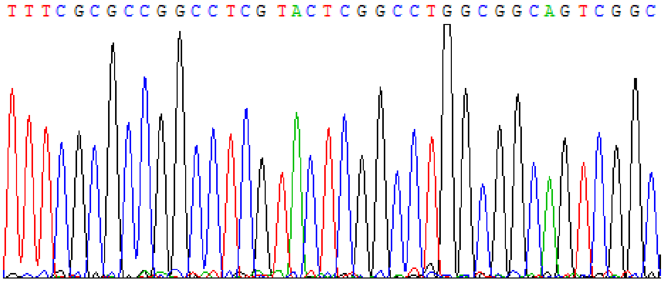

TTTCGCGCCGGCCTCGTACTCGGCCTGGCGGCAGTCGGC   **Type 1(-A)**

TTTCGCGCCGGCCTCGTACTCGGCCATGGCGGCAGTCGGC   **WT**

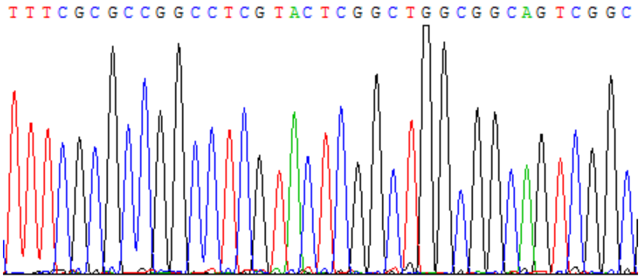

TTTCGCGCCGGCCTCGTACTCGGCTGGCGGCAGTCGGC   **Type 2(-CA)**

TTTCGCGCCGGCCTCGTACTCGGCCATGGCGGCAGTCGGC   **WT**

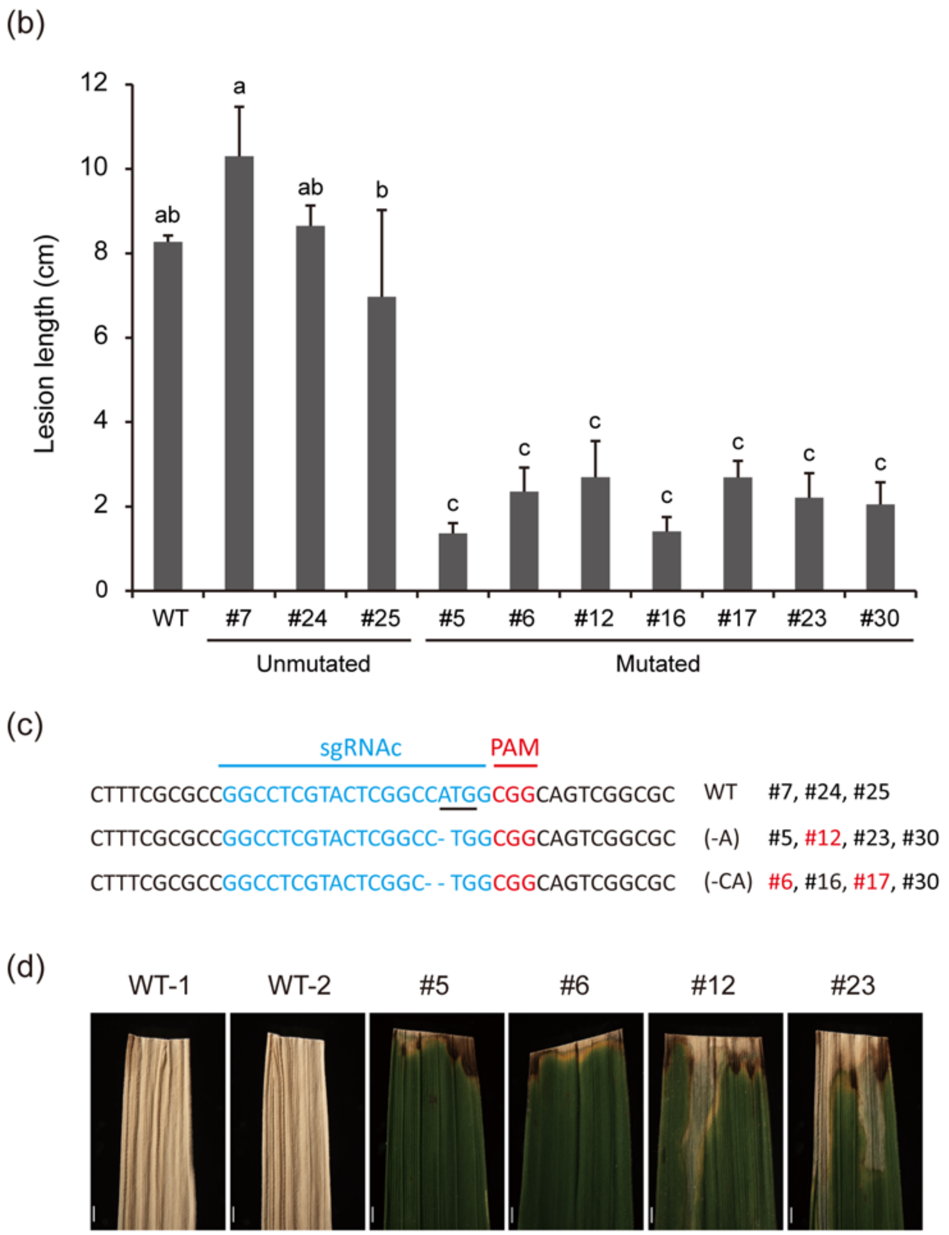

Figure S11 CRISPR/Cas9 plants with the first ATG of *OsWRKY7* mutated had enhanced resistance to *Xoo* and hypersensitive response (HR) related cell death. (a) Detecting mutations by direct sequencing of PCR products containing the sgRNAc targeted site (highlighted in blue color). The nucleotides were read according to the sequencing chromatogram viewed in Chromas and two types of homozygous mutations are shown. The deleted nucleotide is highlighted in yellow on the WT reference and the number is given in brackets for each type. The red letters indicate the PAM site. (b) Lesion lengths on leaves of WT and *OsWRKY7* sgRNAc targeted T_1 lines, which had unmutated or mutated target site shown in (c). Bars represent mean

lesion lengths ± SD (n=3) at 14 dpi. Bars with different lowercase letters are significantly different to each other (one-way ANOVA with Tukey's multiple comparison test, $P<0.05$). (c) Analysis of the mutation types in T_1 siblings from 10 independent lines used in (b). Lines #7, #24, #25 have the WT sequences without mutation at the sgRNAc target. Plants from lines #5, #12, #23, #30 have "A" deletion (-A), and plants from lines #6, #16, #17, #30 have "CA" deletion (-CA). Red numbers are homozygous lines with a single type of mutation in all the T_1 siblings, otherwise, multiple mutated alleles were found in some of the single plants derived from the same T_1 lines. The red letters indicate the PAM site and the ATG site is underlined. (d) *Xoo* infection induced HR-resistant symptoms (dark brown necrosis) on the leaves of the *OsWRKY7* sgRNAc targeted T_1 lines described in (b). Representative leaves from plants of WT and four mutation lines were photographed at 14 dpi. Scale bars, 1 mm.

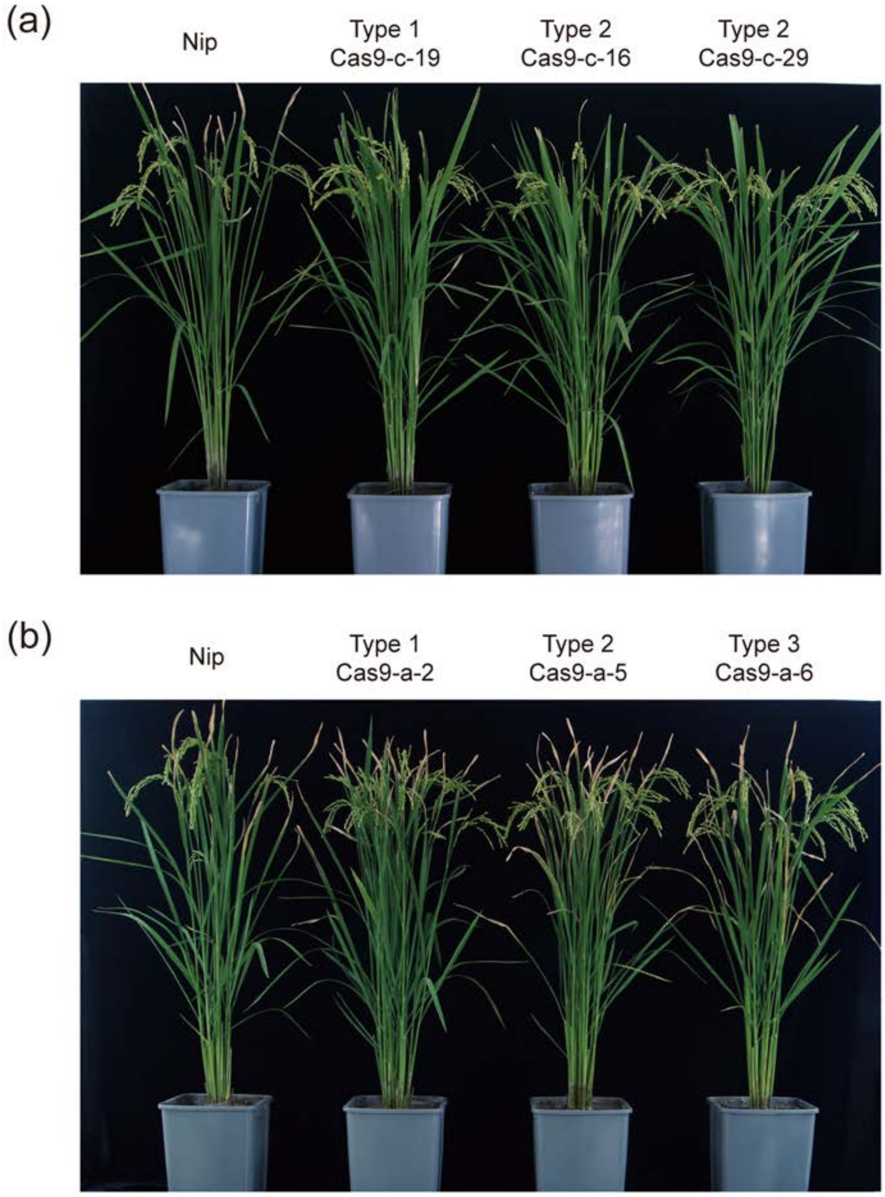

Figure S12 *OsWRKY7* regulated defense response against the highly virulent *Xoo* strain PXO99. (a) Plants with the first ATG of *OsWRKY7* mutated by sgRNAc had high resistance to *Xoo* strain PXO99. Nip control and three homozygous lines from two types of mutation were inoculated with PXO99. (b) Plants with mutation at the end of the first exon of *OsWRKY7* generated by sgRNAa were more susceptible to *Xoo* strain PXO99. Nip control and three homozygous lines from three types of mutation were inoculated with PXO99. The photographs were taken at 14 dpi.

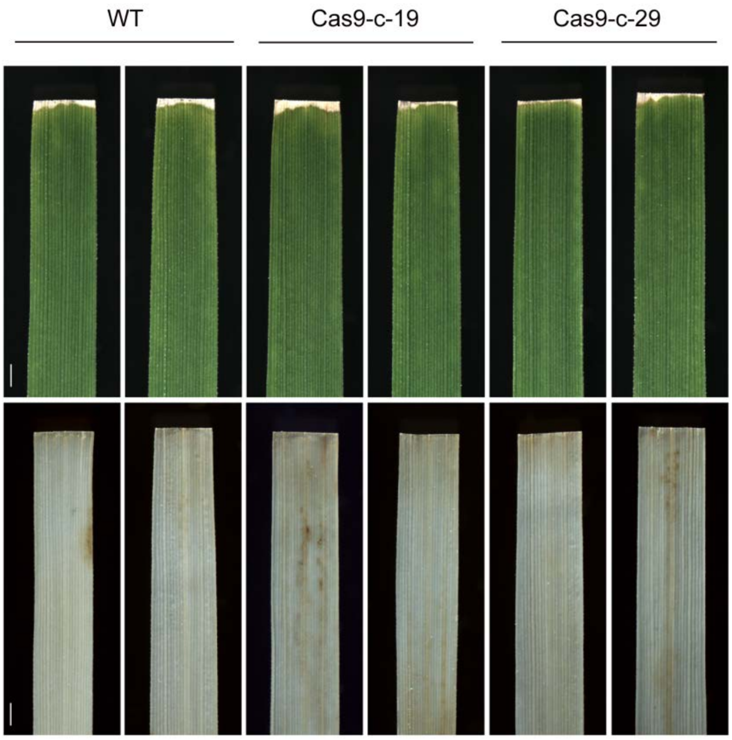

Figure S13 H_2O_2 levels in leaves of WT and *oswrky7-Cas9-c* transgenic plants without *Xoo* infection. Leaves were mock treated with H_2O and photographed at 5 dpi (upper panels). The same leaves were stained with 3,3'-diaminobenzidine (DAB) and photographed under after decoloring (lower panels). Scale bars, 1 mm. Two leaves of each line are shown.

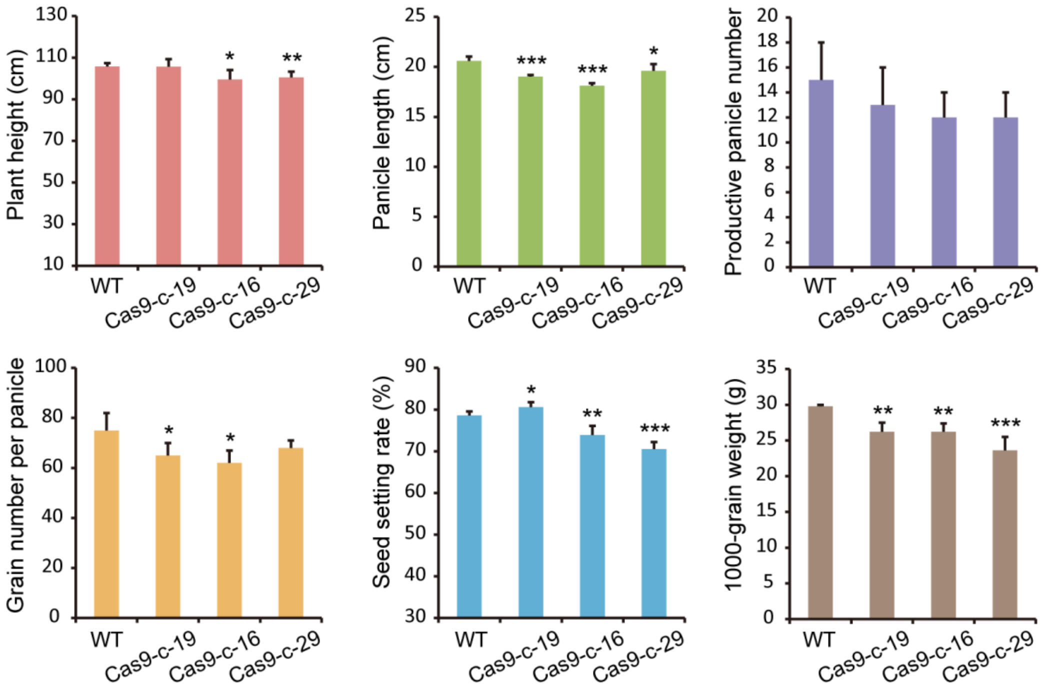

Figure S14 The agronomic phenotypes of the *oswrky7-Cas9-c* transgenic plants.

Agronomic traits in WT and T_2 transgenic lines of *oswrky7-Cas9-c*, including plant height, panicle length, productive panicle number, grain number per panicle, seed setting rate and 1000-grain weight. The data are means±SD (n=4). Significant differences between WT and Cas9 lines are indicated as *P<0.05, **P<0.01, ***P<0.001 by Student's *t*-test.

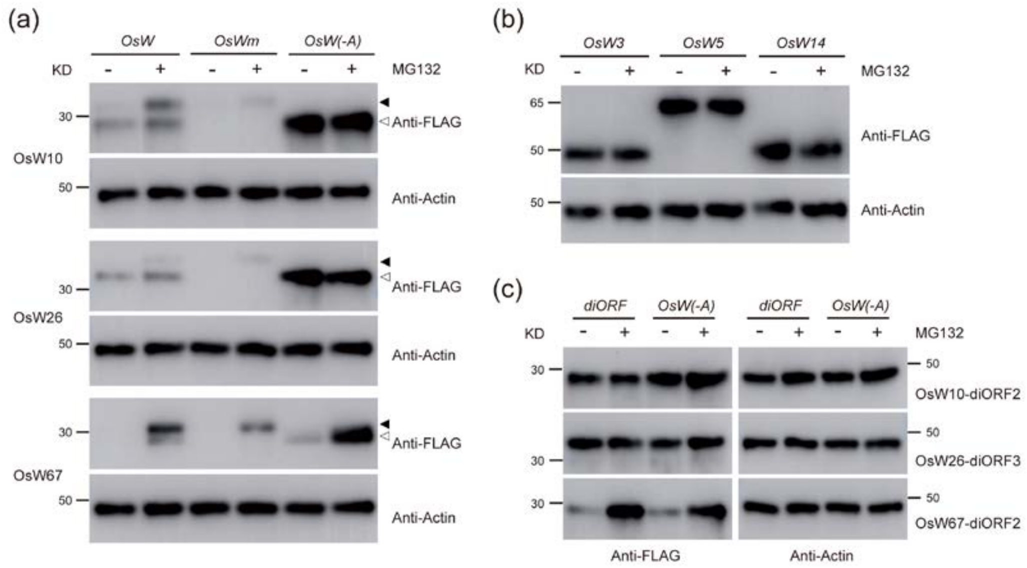

Figure S15 Alternative translation initiation of *OsWRKY* group II members clustering in the clade with *OsWRKY7*. (a) Alternative translation of diORF was also found in *OsWRKY10/26/67* which are group II clade members closely related to *OsWRKY7*. The *OsW* sequence is the full-length CDS. The *OsWm* sequence has a point mutation of the second (*OsWRKY10/67*) or third (*OsWRKY26*) in-frame ATG to AGG. The *OsW(-A)* sequence has a deletion of the first ATG to TG. All the proteins were transiently expressed in rice protoplasts under control of the 35S promoter. Total protein was extracted after treatment with DMSO (-) or 50 μM MG132 (+) and detected by anti-FLAG antibody. The expected full-length and short proteins are indicated with the black and white arrowheads respectively. (b) There was no alternative translation of OsWRKY3/5/14 proteins in rice protoplasts under control of the 35S promoter. (c) Abolishing the first ATG of *OsWRKY10/26/67* enabled the translation of the diORF. The *OsW(-A)* sequence and the second or third diORF were transiently expressed in rice protoplasts under control of the 35S promoter.

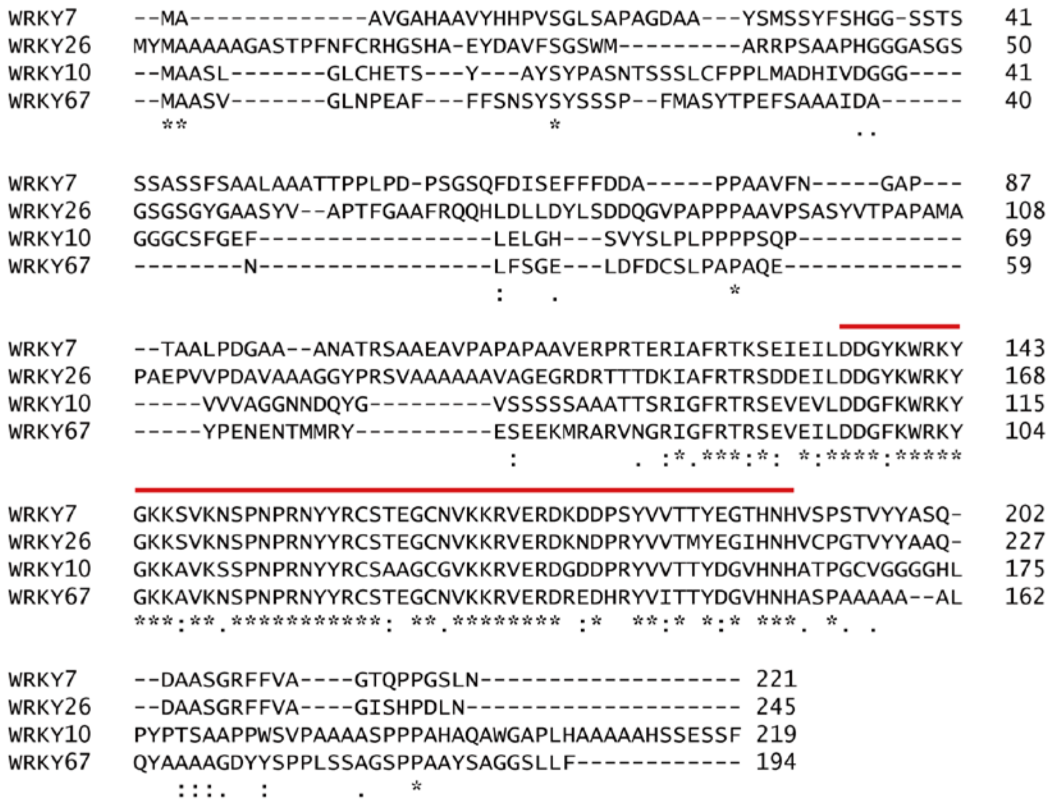

Figure S16 Conservation of OsWRKY7 protein and its closely related homologs. Multiple sequence alignment of OsWRKY7 protein and three other closely-related homologs OsWRKY10, OsWRKY26 and OsWRKY67 by the Clustal Omega program online (https://www.ebi.ac.uk/Tools/msa/clustalo/). The consensus amino acids are shown by "*". The red line indicates the WRKY domain. The sequence identity between OsWRKY7 and OsWRKY26/10/67 was 51.4%, 36.0% and 39.9%, respectively.
