# Supplementary material for: Elevating plant immunity by translational regulation of a rice WRKY transcription factor
Source: Plant Biotechnol J. 2023 Nov 23;22(4):1033–48. doi: 10.1111/pbi.14243 (PMC10955491; doi:10.1111/pbi.14243)
Supplement: Supplementary file 2 — Table S1 Sequences of primers used for vector construction. Table S2. Sequences of primers used for RT‐PCR. Table S3 LC–MS/MS identified peptides in the proteins expressed from the mutated OsWRKY7‐SR gene controlled by 35S promoter. Table S4. Comparison of the AUG initiation codon context (−6 to +4) in the OsWRKY genes tested in this study. [file PBI-22-1033-s003.doc]

**Table S1** Sequences of Primers Used for Vector Construction

| **No.** | **Primer Name** | **Primer Sequence (5’-3’)** | **Note** |
| --- | --- | --- | --- |
| 1 | sgRNAa-F | GCCGCGCCGCCGCAAACGCAACA | To construct CRISPR/Cas9 vector for sgRNAa target site under U6a promoter |
| 2 | sgRNAa-R | AAACTGTTGCGTTTGCGGCGGCG |
| 3 | sgRNAb-F | GGCATGTCATCGTACTTCTCCCA | To construct CRISPR/Cas9 vector for sgRNAb target site under U3 promoter |
| 4 | sgRNAb-R | AAACTGGGAGAAGTACGATGACA |
| 5 | sgRNAc-F | GCCGGCCTCGTACTCGGCCATGG | To construct CRISPR/Cas9 vector for sgRNAc target site under U6a promoter |
| 6 | sgRNAc-R | AAACCCATGGCCGAGTACGAGGC |
| 7 | Cas9-a-F | GCGACGCCGCCTACTCGA | To amplify the fragment of sgRNAa target site for sequencing |
| 8 | Cas9-a-R | CCGCCGCGCTCCTGAAT |
| 9 | Cas9-b/c-F | ATCACGATCGATCTCATCCCTCT | To amplify the fragment of sgRNAb/c target sites for sequencing |
| 10 | Cas9-b/c-R | CGCGTCGTCGAAGAAGAACTCGGA |
| 11 | HPT-F | CGCCGATGGTTTCTACAAAG | To amplify the fragment of *HPT* gene |
| 12 | HPT-R | ACACATGGGGATCAGCAATC |
| 13 | OsW7-FL-F+T | tATGGCGGCAGTCGGCGCG | To amplify the full-length CDS of *OsW7* gene for T-A cloning into pXB2E entry vector |
| 14 | OsW7-FL-R+T | tTTAATTGAGAGAACCTGGCGGCTGCGTG |
| 15 | OsW10-FL-F | ATGGCGGCTTCGCTGGGAC | To amplify the full-length CDS of *OsW10* gene |
| 16 | OsW10-FL-R | TCAGAACGACGATTCCGACGAGT |
| 17 | OsW26-FL-F | CGTGTACCGATGTACATGGC | To amplify the full-length CDS of *OsW26* gene |
| 18 | OsW26-FL-R | ATTAAGGTCTGGGTGACTGAT |  |
| 19 | OsW67-FL-F | ATGGCGGCTTCCGTAGGACTGA | To amplify the full-length CDS of *OsW67* gene |
| 20 | OsW67-FL-R | TCAGAAGAGCAGCGAGCCGCCTG |
| 21 | OsW7-FL-FW-1 | cggtacccggggatccATGGCGGCAGTCGGCGCG | To construct 35S-OsW7-3xflag vector by infusion |
| 22 | OsW7-FL-RV-1 | gggcgaattggtcgacATTGAGAGAACCTGGCGGC |
| 23 | OsW7-NT1-RV-1 | gggcgaattggtcgacGTTTGCGGCGGCGCCATCC | To construct 35S-NT1-3xflag vector by infusion, pair with OsW7-FL-FW-1 |
| 24 | OsW7-CT1-FW-1 | cggtacccggggatccATGGCAACAAGGAGCGCG | To construct 35S-CT1-3xflag vector by infusion, pair with OsW7-FL-RV-1 |
| 25 | OsW7-CT4-FW-1 | cggtacccggggatccATGTCATCGTACTTCTCCCACGG | To construct 35S-CT4-3xflag vector by infusion, pair with OsW7-FL-RV-1 |
| 26 | OsW10-FL-FW-1 | cggtacccggggatccATGGCGGCTTCGCTGGGAC | To construct 35S-OsW10-3xflag vector by infusion |
| 27 | OsW10-FL-RV-1 | gggcgaattggtcgacGAACGACGATTCCGACGAG |
| 28 | OsW26-FL-FW-1 | cggtacccggggatccATGTACATGGCGGCGGC | To construct 35S-OsW26-3xflag vector by infusion |
| 29 | OsW26-FL-RV-1 | gggcgaattggtcgacATTAAGGTCTGGGTGACTGAT |
| 30 | OsW67-FL-FW-1 | cggtacccggggatccATGGCGGCTTCCGTAGGACTGA | To construct 35S-OsW67-3xflag vector by infusion |
| 31 | OsW67-FL-RV-1 | gggcgaattggtcgacGAAGAGCAGCGAGCCGCCTG |
| 32 | OsW7-FL-FW-3 | gctaattcgagctcggtaccggatccATGGCGGCAGTC | To construct Ubi-OsW7-3xflag vector by infusion |
| 33 | OsW7-FL-RV-3 | cgactctagaggatcTCATTTGTCATCATCGTCTTTGTAG |
| 34 | OsW7-FL-FW-GST | tggatccccggaattcATGGCGGCAGTCGGCGCG | To construct GST-OsW7 vector by infusion |
| 35 | OsW7-FL-RV-GST | ggccgctcgagtcgacTTAATTGAGAGAACCTGGCG |
| 36 | OsW7-FL-FW-GFP | tcgagctcggtacccATGGCGGCAGTCGGCGCG | To construct Ubi-OsW7-GFP vector by infusion |
| 37 | OsW7-FL-RV-GFP | cactagtggatcccccATTGAGAGAACCTGGCGGC |
| 38 | OsW7-NT1-RV-GFP | cactagtggatccccCGTTTGCGGCGGCGCCATCC | To construct Ubi-NT1-GFP vector by infusion, pair with OsW7-FL-FW-GFP |
| 39 | OsW7-CT1-FW-GFP | tcgagctcggtacccATGGCAACAAGGAGCGCG | To construct Ubi-CT1-GFP vector by infusion, pair with OsW7-FL-RV-GFP |
| 40 | OsW7-CT4-FW-GFP | tcgagctcggtacccATGTCATCGTACTTCTCCCACGG | To construct Ubi-CT4-GFP vector by infusion, pair with OsW7-FL-RV-GFP |
| 41 | OsW7-FL-FW-BD | aggaggacctgcatatgGCGGCAGTCGGCGCG | To construct pGBKT7-OsW7 vector by infusion |
| 42 | OsW7-FL-RV-BD | gcaggtcgacggatcTTAATTGAGAGAACCTGGCG |
| 43 | OsW7-NT1-RV-BD | gcaggtcgacggatccGTTTGCGGCGGCGCCATCC | To construct pGBKT7-OsW7-NT1 vector by infusion, pair with OsW7-FL-FW-BD |
| 44 | OsW7-NT2-RV-BD | gcaggtcgacggatccAATCTCGATCTCTGACTTCGTCCGG | To construct pGBKT7-OsW7-NT2 vector by infusion, pair with OsW7-FL-FW-BD |
| 45 | OsW7-NT3-RV-BD | gcaggtcgacggatccCACGTGGTTGTGCGTCCC | To construct pGBKT7-OsW7-NT3 vector by infusion, pair with OsW7-FL-FW-BD |
| 46 | OsW7-CT1-FW-BD | aggaggacctgcatatgGCAACAAGGAGCGCGGCG | To construct pGBKT7-OsW7-CT1 vector by infusion, pair with OsW7-FL-RV-BD |
| 47 | OsW7-CT2-FW-BD | aggaggacctgcatatgGACGCGCCTCCGGCCGCC | To construct pGBKT7-OsW7-CT2 vector by infusion, pair with OsW7-FL-RV-BD |
| 48 | OsW7-CT3-FW-BD | aggaggacctgcatatgCTCGCCGCCGCAACGACG | To construct pGBKT7-OsW7-CT3 vector by infusion, pair with OsW7-FL-RV-BD |
| 49 | OsW7-CT4-FW-BD | aggaggacctgcatatgTCATCGTACTTCTCCCACGG | To construct pGBKT7-OsW7-CT4 vector by infusion, pair with OsW7-FL-RV-BD |
| 50 | OsW7-ORF2-FW | cggtacccggggatccATGTCATCGTACTTCTC | To construct 35S-OsW7-ORF2-3xflag vector by infusion, pair with OsW7-FL-RV-1 |
| 51 | OsW10-ORF2-FW | cggtacccggggatccATGGCGGACCACATCGTCG | To construct 35S-OsW10-ORF2-3xflag vector by infusion, pair with OsW10-FL-RV-1 |
| 52 | OsW26-ORF3-FW | cggtacccggggatccATGGCGCGCCGCCCCT | To construct 35S-OsW26-ORF3-3xflag vector by infusion, pair with OsW26-FL-RV-1 |
| 53 | OsW67-ORF2-FW | cggtacccggggatccATGGCCAGCTACACGCCGGAG | To construct 35S-OsW67-ORF2-3xflag vector by infusion, pair with OsW67-FL-RV-1 |
| 54 | OsW7-(-A)-FW | cggtacccggggatccTGGCGGCAGTCGGCGCG | To construct 35S-OsW7-(-A)-3xflag vector by infusion, pair with OsW7-FL-RV-1 |
| 55 | OsW10-(-A)-FW | cggtacccggggatccTGGCGGCTTCGCTGGGAC | To construct 35S-OsW10-(-A)-3xflag vector by infusion, pair with OsW10-FL-RV-1 |
| 56 | OsW26-(-A)-FW | cggtacccggggatccTGTACATGGCGGCGGC | To construct 35S-OsW26-(-A)-3xflag vector by infusion, pair with OsW26-FL-RV-1 |
| 57 | OsW67-(-A)-FW | cggtacccggggatccTGGCGGCTTCCGTAGGACTGA | To construct 35S-OsW67-(-A)-3xflag vector by infusion, pair with OsW67-FL-RV-1 |
| 58 | OsW7-muta-F | GCCGCCTACTCGAGGTCATCGTACTTCTCC | To substitute the T in ATG of OsW7 with G |
| 59 | OsW7-muta-R | GGAGAAGTACGATGACCTCGAGTAGGCGGC |
| 60 | OsW10-muta-F | GTGCTTCCCTCCTCTCAGGGCGGACCA | To substitute the T in ATG of OsW10 with G |
| 61 | OsW10-muta-R | TGGTCCGCCCTGAGAGGAGGGAAGCAC |
| 62 | OsW26-muta-F | TCAGCGGCAGCTGGAGGGCGCGCC | To substitute the T in ATG of OsW26 with G |
| 63 | OsW26-muta-R | GGCGCGCCCTCCAGCTGCCGCTGA |
| 64 | OsW67-muta-F | CCTACTCCTCATCCCCTTTCAGGGCCAGCTACA | To substitute the T in ATG of OsW67 with G |
| 65 | OsW67-muta-R | TGTAGCTGGCCCTGAAAGGGGATGAGGAGTAGG |  |
| 66 | OsW7-pr-gDNA-FW | acgaattcgagctcggtaccCGACGCCCTATCCGATTCC | To amplify OsW7 promoter fragment for overlapping |
| 67 | OsW7-pr-gDNA-R | CATATATTATCGATGTTTTTAAATACGCACCCGAC |
| 68 | OsW7-pr-(-A)gDNA-R | CGCGCCGACTGCCGCCAGGCCGAGTACGAGGCCGG | To amplify OsW7 promoter fragment (without A of ATG) for overlapping, pair with OsW7-pr-gDNA-FW |
| 69 | OsW7-pr-gDNA-muta-R | TGGGAGAAGTACGATGACCTCGA | To amplify OsW7 gDNA with mutation in the second ATG for overlapping, pair with OsW7-pr-gDNA-FW |
| 70 | OsW7-pr-gDNA-RV | gggcgaattggtcgacATTGAGAGAACCTGGCGGC | To amplify part of OsW7 promoter and gDNA fragment for overlapping |
| 71 | OsW7-pr-gDNA-F | GTCGGGTGCGTATTTAAAAACATCGATAATATATG |
| 72 | OsW7-pr-(-A)gDNA-F | CCGGCCTCGTACTCGGCCTGGCGGCAGTCGGCGCG | To amplify OsW7 gDNA fragment (without A of ATG) for overlapping, pair with OsW7-pr-gDNA-RV |
| 73 | OsW7-pr-gDNA-muta-F | TCGAGGTCATCGTACTTCTCCCA | To amplify OsW7 gDNA with mutation in the second ATG for overlapping, pair with OsW7-pr-gDNA-RV |
| 74 | GFP-Myc-FW | ggggatcctctagagtcgacATGGTGAGCAAGGGCGAGG | To construct 35S-Myc-GFP vector by infusion |
| 75 | GFP-Myc-RV | atgtttgaacgatcctgcagTTACTTGTACAGCTCGTCCA |
| 76 | UbL40-F | ATGCAGATCTTCGTGAAGA | To amplify ubiquitin (1-228 bp) from *UbL40* (Os09g0452700) gene |
| 77 | UbL40-R | GCCACCGCGGAGGCG |
| 78 | UbL40-Myc-FW | ggggatcctctagagtcgacATGCAGATCTTCGTGAAGA | To construct 35S-Myc-Ubi vector by infusion |
| 79 | UbL40-Myc-RV | atgtttgaacgatcctgcagTCAGCCACCGCGGAG |
| 80 | H2B-mCherry-FW | cgggggacgagctcggtaccATGGCGAAGGCAGATAAGAA | To construct 35S-H2B-mCherry vector by infusion |
| 81 | H2B-mCherry-RV | tgctcaccatgtcgacAGAACTCGTAAACTTCGTAACC |
| 82 | OsW7-pr-gDNA-FW-2 | gatcccccgaattagagctcCGACGCCCTATCCGATTCC | To amplify OsW7-pr-gDNA/(-A)/muta-3xFLAG-rbcs for infusion into the CSP1-HPT-MCS-GUS vector |
| 83 | Ter-RV | actcctcttaaagcttGATGCATGTTGTCAATCAATTGG |
| 84 | OsW7-pr-gDNA-FW-3 | atagggcgaattgggtaccCGACGCCCTATCCGATTCC | To amplify OsW7-pr-N81/N81(-A) fragments for infusion into pGreen-0800-Luc vector. |
| 85 | OsW7-N81-Luc-RV | ttttggcgtcttccatCGAGTAGGCGGCGTCGCC |
| 86 | WRKY7-SR-1F | ATGGCGGCAGTCGGCGCGCACGCAGCGGTCTACCACCACCCGGTCAGGGGCCTCTCGGCGCCGGCGGGCGACGCC | To generate *WRKY7-SR* adaptor 1 with S16R mutation for overlapping with adaptor 2 |
| 87 | WRKY7-SR-1R | GGCGTCGCCCGCCGGCGCCGAGAGGCCCCTGACCGGGTGGTGGTAGACCGCTGCGTGCGCGCCGACTGCCGCCAT |
| 88 | WRKY7-SR-2F | CGGGCGACGCCGCCTACAGGATGTCATCGTACTTCTCCCACGGGGGAAGCTCGACCTCCAGCCGCGCGTCCAGCT | To generate *WRKY7-SR* adaptor 2 with S28R and S43R mutations for overlapping with adaptor 1 |
| 89 | WRKY7-SR-2R | AGCTGGACGCGCGGCTGGAGGTCGAGCTTCCCCCGTGGGAGAAGTACGATGACATCCTGTAGGCGGCGTCGCCCG |
| 90 | WRKY7-SR-3F | TCCAGCCGCGCGTCCAGCTTCT | To amplify rest part of *WRKY7-SR* (122-663 bp) for overlapping with adaptor 1+2, pair with OsW7-FL-RV-1 |

**Table S2** Sequences of Primers Used for RT-PCR

| **Primer Name** | **UPL** | **Primer Sequence (5’-3’)** |
| --- | --- | --- |
| OsWRKY7-UPL-F | #105 | TGATCGAGATAGCAGCCTCTT |
| OsWRKY7-UPL-R | #105 | CCAGCTACATCCAAAATGACC |
| OsActin1-UPL-F | #158 | CAACACCCCTGCTATGTACG |
| OsActin1-UPL-R | #158 | CATCACCAGAGTCCAACACAA |
| OsPR1a-RT-F | - | TCACACTCACTAATTAATCATA |
| OsPR1a-RT-R | - | CAAATACGGCTGACAGTACA |
| OsPR1b-RT-F | - | ACGCCTTCACGGTCCATAC |
| OsPR1b-RT-R | - | AAACAGAAAGAAACAGAGGGAGTAC |
| OsPR5-RT-F | - | CAGCCAGGACTTCTACGACC |
| OsPR5-RT-R | - | CATGAGATGATGCATTATGGG |
| OsPR10a-RT-F | - | GGGCACCATCTACACCATGAA |
| OsPR10a-RT-R | - | TCGTACTCCACCTTGAGCTT |
| OsRbohB-RT-F |  | TCGCTGGTATTTGAAAACGAC |
| OsRbohB-RT-R |  | TCCACTTCTATATCGAAATCCGTA |
| OsRbohE-RT-F |  | TCAGATCCAAGGCTTATGCAG |
| OsRbohE-RT-R |  | TTGGCACGAAATAGTTCTCGAC |
| OsActin1-RT-F |  | GAGTATGATGAGTCGGGTCCAG |
| OsActin1-RT-R |  | ACACCAACAATCCCAAACAGAG |

**Table S3 LC-MS/MS Identified Peptides in the Proteins Expressed from the Mutated *OsWRKY7-SR* Gene Controlled by 35S Promoter**

| **Peptide Sequence^a^** | **#PSMs** | **MH+ [Da]** | **Abundances** | **DeltaM [ppm]** | **RT [min]** | **Ions Score** |
| --- | --- | --- | --- | --- | --- | --- |
| **OsWRKY7-SR-MG132-up** |  |  |  |  |  |  |
| MSSYFSHGGSSTSSR | 2 | 1593.66 | 5.20E+06 | 0.17 | 8.36 | 73 |
| DKDDPSYVVTTYEGTHNHVSPSTVYYASQDAASGR | 3 | 3817.72 | 7.03E+06 | 0.65 | 15.50 | 72 |
| SEIEILDDGYK | 1 | 1281.62 | 7.45E+06 | 0.2 | 16.70 | 63 |
| AAVGAHAAVYHHPVR | 2 | 1597.83 | 6.13E+06 | -0.38 | 10.16 | 57 |
| SAAEAVPAPAPAAVERPR | 1 | 1759.94 | 1.06E+07 | -1.06 | 12.69 | 43 |
| GLSAPAGDAAYR | 1 | 1148.57 | 1.67E+07 | 0.25 | 11.47 | 41 |
| **OsWRKY7-SR-MG132-down** |  |  |  |  |  |  |
| DKDDPSYVVTTYEGTHNHVSPSTVYYASQDAASGR | 3 | 3817.72 | 3.13E+07 | 0.65 | 15.55 | 60 |
| SEIEILDDGYK | 1 | 1281.62 | 1.88E+07 | 0.01 | 16.70 | 58 |
| TKSEIEILDDGYK | 1 | 1510.76 | 6.54E+06 | 0.53 | 14.10 | 48 |
| SAAEAVPAPAPAAVERPR | 2 | 1759.94 | 8.05E+07 | -0.95 | 12.69 | 42 |
| **OsWRKY7-SR-DMSO-down** |  |  |  |  |  |  |
| SEIEILDDGYK | 1 | 1281.62 | 3.01E+07 | 0.2 | 16.60 | 66 |
| DKDDPSYVVTTYEGTHNHVSPSTVYYASQDAASGR | 2 | 3817.72 | 3.14E+07 | 0.09 | 15.55 | 58 |
| SAAEAVPAPAPAAVERPR | 1 | 1759.94 | 2.13E+07 | 0.23 | 12.70 | 41 |
| TKSEIEILDDGYK | 1 | 1510.76 | 1.28E+06 | 1.58 | 14.71 | 41 |

**a** Identified peptide sequence that target to the OsWRKY7-SR protein which has S16R, S28R and S43R mutations.

**#PSMs**: the total number of identified peptide spectrum matches for the protein; **MH+ [Da]**: calculated m/z of the peptide with z = 1; **Abundances**: ion abundances of a peptide; **DeltaM [ppm]**: mass measurement error in parts per million, ppm; **RT [min]**: the peptide’s retention time during chromatographic separation; **Ions Score**: a measure of how well the observed MS/MS spectrum matches to the stated peptide.

**Table S4** Comparison of the AUG Initiation Codon Context (-6 to +4) in *OsWRKY* Genes Tested in this Study

| Gene | Sequence (-6 to +4) | Consensus (-3/+4) | Category^a^ |
| --- | --- | --- | --- |
| *OsWRKY7* | UCGGCCAUGG | G/G | Strong |
| *OsWRKY10* | GCGAGCAUGG | A/G | Strong |
| *OsWRKY26* | GUACCGAUGU | C/U | Weak |
| *OsWRKY67* | UGUGCAAUGG | G/G | Strong |
| *OsWRKY3* | GCGGCCAUGG | G/G | Strong |
| *OsWRKY5* | CUAUCCAUGG | U/G | Adequate |
| *OsWRKY14* | AUAUAUAUGG | U/G | Adequate |

a Sequences surroungding the AUG initiation codon were categorized depending on the presence of the two crucial nucleotides at -3 and +4 within the Kozak motif: ‘Optimal’, GCC*R*CCAUGG; ‘Strong’, NNN*R*NNAUGG; ‘Adequate’, NNN*R*NNAUG(A/C/U) or NNN(C/U)NNAUGG; ‘Weak’, NNN(C/U)NNAUG(A/C/U). *R* is A or G, N is any base, AUG initiation codon is underlined.
