# Supplementary material for: Elevating plant immunity by translational regulation of a rice WRKY transcription factor
Source: Plant Biotechnol J. 2023 Nov 23;22(4):1033–48. doi: 10.1111/pbi.14243 (PMC10955491; doi:10.1111/pbi.14243)
Supplement: Supplementary file 3 — Data S1 Supporting Information. [file PBI-22-1033-s001.doc]

**Vector construction and plant transformation**

The *OsWRKY7* genome-edited constructs were generated by the method previously described (Ma et al., 2015). Three target sequences (No.1-6) at different genome sites (a, b, and c) were introduced into *pYLgRNA* vectors under the rice *U6a* or *U3* promoters, then the single sgRNA expression cassette was ligated into the binary vector *pYLCRISPR/Pubi-H*. Mutations in transgenic plants were detected by PCR amplification using the primer pairs (No.7-10) flanking the designed target sites and sequenced directly. Transgenic plants lacking the T-DNA insertion were identified by PCR amplification of the hygromycin gene (No.11-12).

To generate the *35S::OsWRKY7/10/26/67-3*×*FLAG* constructs, the full-length CDS (without TAA) of *OsWRKY7/10/26/67* was first amplified with specific primers (No. 13-20) and re-amplified with adaptor primers (No.21,22,26-31) for C-terminal fusion with 3×FLAG in the *35S-3*×*FLAG-1300* vector digested with *BamH*I and *Sal*I using the In-Fusion HD Cloning kit (Takara, Otsu, Japan). Similarly, the forward adaptor primers (50-53) were used to generate *35S::OsWRKY7/10/26/67-diORF-3*×*FLAG* and the forward adaptor primers (No.54-57) excluding A of ATG were used to generate *35S::OsWRKY7/10/26/67-(-A)-3*×*FLAG* by In-Fusion cloning. The *35S::OsWRKY7-(-A)-3*×*FLAG* was used for rice transformation. To generate the *35S:: OsWRKY7/10/26/67-muta-3*×*FLAG* constructs, the second or third in-frame ATG in *35S::OsWRKY7/10/26/67-3*×*FLAG* was mutated to AGG by site-specific mutation primers (No.58-65) with the QuikChange Lightning Site-Directed Mutagenesis kit (Agilent Technologies, Santa Clara, CA). To generate the *35S::OsWRKY7-SR-3*×*FLAG* vector for LC-MS/MS analysis, adaptor primers (No.86-89) were designed to mutate the Ser (CTC) at site 16, 28 and 43 to Arg (CAG or CCG) by overlapping PCR. The remaining part of *OsWRKY7-SR* (122-663 bp) was amplified by primers (No.90,22). The whole *OsWRKY7-SR* was amplified with adaptor primers (No.21-22) for fusion with 3×FLAG in the *35S-3*×*FLAG-1300* vector digested with *BamH*I and *Sal*I.

To generate the *Ubi::OsWRKY7-3*×*FLAG* constructs for expression in protoplasts and transgenic plants, the *OsWRKY7-3*×*FLAG* fragments were amplified from the *35S::OsWRKY7-3×FLAG* plasmid with adaptor primers (No.32-33) for In-Fusion cloning into the *Ubi-1300* vector digested with *Kpn*I and *BamH*I.

To generate the *GST-OsWRKY7* construct for recombinant protein expression, the full-length CDS (without TAA) of *OsWRKY7* was amplified with adaptor primers (No. 38-39) for cloning into the *pGEX-4T-1* vector digested with *EcoR*I and *Sal*I.

To generate the *pGBKT7-OsWRKY7/NT1/NT2/NT3/CT1/CT2/CT3/CT4* constructs for yeast expression, the full-length CDS of *OsWRKY7* (1-666 nt), N-terminal fragment NT1 (1-294 nt), NT2 (1-399 nt), NT3 (1-576 nt), and C-terminal fragment CT1 (295-666 nt), CT2 (226-666 nt), CT3 (151-666 nt), CT4 (85-666 nt) were amplified with adaptor primers (No.41-49) for In-Fusion cloning into *pGBKT7* (Clontech, CA, USA) digested with *Nde*I and *BamH*I.

To generate the *Ubi::OsWRKY7/NT1/CT1/CT4-GFP* constructs for protein localization, the fragments of full-length *OsWRKY7*, NT1, CT1 and CT4 were amplified with adaptor primers (No.36-40) for C-terminal fusion to GFP in the *Ubi-GFP-1300* vector digested with *Sma*I. To generate the nucleus marker, the *Arabidopsis* histone *H2B* gene (AT5G22880) (Federici et al., 2012) was amplified with adaptor primers (No.80-81) from a vector containing *H2B*, and fused to the C-terminal mCherry in the *35S-mCherry-1300* vector digested with *Sal*I and *Pst*I.

To generate the *pOsWRKY7::OsW7--3*×*FLAG* construct, a ~4-kb fragment containing genomic *OsWRKY7* (without TAA) and ~3-kb region upstream of the start codon was amplified from genomic DNA of *Nipponbare* using overlapping PCR (No.66-67,70-71) and then fused at the C-terminal with 3xFLAG in the *3*×*FLAG-1300* vector digested with *Kpn*I and *Sal*I. Overlapping PCR primers (No.68,72 and No. 69,73 ) were designed to amplify the *pOsWRKY7::OsW7(-A)* and *pOsWRKY7::OsW7m* fragment from the plasmid of *pOsWRKY7::OsW7-3*×*FLAG* and then cloned into the *3*×*FLAG-1300* vector for protoplast expression. To generate transgenic plants, both the *pOsWRKY7::OsW7-3*×*FLAG-rbcs* and *pOsWRKY7::OsW7(-A)-3*×*FLAG-rbcs* fragments were amplified with adaptor primers (No.82-83) and cloned into a modified binary vector *CSP1-HPT-MCS-GUS-Tnos* (Zhou et al., 2020) to replace *GUS-Tnos* by *Sac*I and *Hind*III digestion. For construct *pOsWRKY7::N81-Luc and pOsWRKY7::N81(-A)-Luc* vectors, fragments of *OsWRKY7* promoter and N terminal 81 bp or 80 bp without A of the first ATG were amplified with adaptor primers (No.84-85) and cloned into *pGreen-0800-Luc* digested with *Kpn*I and *Nco*I by In-Fusion.

To generate the *35S::Myc-GFP* and *35S::Myc-Ubi* constructs, the coding sequence of GFP was amplified with adaptor primers (No.74-75) for N-terminal fusion to Myc in the *35S-Myc-1300* vector digested with *Sal*I and *Pst*I. The coding sequence of ubiquitin was amplified from the *UbL40* (Os09g0452700) gene (1-228 nt) with specific primers (No.76-77) and re-amplified with adaptor primers (No.78-79) for N-terminal fusion to Myc in the same *35S-Myc* *-1300* vector.

The DNA polymerase KOD-Fx (Toyobo, Osaka, Japan) was used for fragment amplification. The restriction enzymes were obtained from NEB (New England Biolabs, Ipswich, MA). All primers used for plasmid construction are listed in the Table S1.

**RNA isolation and qRT-PCR analysis**

Total RNAs were extracted by TRIzol Reagent (Invitrogen, Carlsbad, CA). 0.5 μg of total RNA of each sample was reverse transcribed by the iScript cDNA Synthesis Kit (Bio-Rad, Mississauga, Canada). qRT-PCR was performed using the Probe master kit or SsoFast EvaGreen Supermix kit (Bio-Rad, Mississauga, Canada) on the LightCycler 480 instrument (Roche, Basle, Switzerland). The relative mRNA transcript levels were normalized to *Actin* and calculated by the 2 ^−ΔΔCt^ method (Pfaffl, 2001). The primers and UPL probes used for qRT-PCR analysis are listed in Table S2.

**Protein extraction and immunoblotting**

Total proteins from 100 μL rice protoplasts or 20 mg leaf samples were extracted with 0.2 mL of denaturing lysis buffer (50 mM Tris-HCl, pH 7.5, 150 mM NaCl, 4 M Urea, 0.1% Nonidet P40, 1 mM PMSF and 1×plant protease inhibitor cocktail from Thermo Scientific). Proteins were separated by 4-12% Bis-Tris PAGE Gel (GenScript, Nanjing, China) and transferred onto nitrocellulose membrane (Merck Millipore, Ireland), then analyzed by immunoblotting with anti-FLAG (1:10000, GenScript, A00187) primary antibody and the secondary antibody (1:5000, Abbkine, A21010). Plant Actin protein detected with anti-Actin antibody (1:10000, Abbkine, A01050) or rubisco large protein (RubL) stained by Coomassie blue were used as loading controls. The relative intensity of protein bands was quantified using ImageJ software.

**Protein subcellular localization**

GFP-tagged OsWRKY7, NT1, NT4 and CT3 proteins were expressed in rice protoplasts under control of the *Ubi* promoter. A H_2_B-mCherry vector was co-expressed as the nucleus marker. The fluorescence signal was visualized using a confocal laser scanning microscope (Zeiss LSM880) under 488 nm and 543 nm laser excitation. Fluorescence was detected at 493-587 nm for GFP, and 578-630 nm for mCherry.

**Transcriptional activity assay in yeast**

The transcriptional activity assay was performed using the Matchmaker Gold Yeast Two-Hybrid System (Clontech). *pGBKT7* vectors containing the full-length or a series of C- and N-terminal truncated *OsWRKY7* fragments were transformed into Y2HGold yeast cells. The transformed yeast cells were grown on selective SD/-Trp medium at 30°C for 3-4 days. About 5-10 colonies were mixed in 100 μL ddH_2_O and transferred as one sample (8 μL) onto SD/-Trp/ X-a-Gal medium. Three samples for each transformation were loaded and the empty *pGBKT7* vector was used as a negative control.

**In vivo phosphorylation assay**

Lamda protein phosphatase (λ-PPase) treatment was performed according to the manufacturer’s instruction (NEB, P0753) with slight modification. Five tubes of rice protoplasts were transfected with *35S::OsWRKY7-3×FLAG* for 12 h. Total protein was extracted from each tube with 100 μL λ-PPase buffer (1×NEB PMP buffer, 1 mM MnCl_2_, 1% Triton X-100, 1×plant protease inhibitor cocktail from Thermo Scientific). Protein extracts after centrifugation (13000 rpm for 10 min) were treated with 1 μL λ-PPase at 30°C for 15 min, 30 min, 45 min and 60 min. The reactions were stopped by 5×SDS loading buffer and subsequently subjected to immunoblot analysis with anti-FLAG antibody. Sample in λ-PPase buffer without phosphatase was used as mock control.

**LC-MS/MS analysis**

Rice protoplasts expressing *35S::OsWRKY7-SR-3×FLAG* were treated with DMSO (8 tubes) and 50 μM MG132 (8 tubes) for 12 h. Total proteins from each tube were extracted in 100 μL IP lysis buffer (Pierce, Thermo Scientitic) plus 1×plant protease inhibitor cocktail (Thermo Scientitic) and extracts in the same treatment were combined. OsWRKY7-SR-FLAG protein was then precipitated by incubation with an anti-FLAG magnetic beads (Genscript, L00790-1) at 4°C for 2 h. The immunoproteins were washed four times with 1×PBS (5 mM DTT) and eluted with 70 μL 2×SDS loading buffer by heating at 100 °C for 10 min. 7 μL eluted sample from each treatment was subjected to western blot analysis with anti-FLAG antibody. The rest of samples were separated by 15% Bis-Tris PAGE Gel (GenScript, China) and stained with Coomassie Brilliant Blue R-250. Gel strips containing the upper or lower band of FLAG tagged OsWRKY7-SR protein were cut out according to their corresponding size indicated in western blot and sent to Jingjie Ptm BioLab (Hangzhou, China) Co. Ltd.

After in-gel trypsin digestion, peptides were subjected to LC-MS/MS analysis using an EASY-nLC 1000 ultra-high pressure liquid chromatography system (Thermo Scientific) connected to tandem mass spectrometry (MS/MS) in Q Exactive^TM^ Plus (Thermo Scientific) with a nanospray ionization source. The resulting MS/MS data were processed using Proteome Discoverer 2.4 to search a database encompassing Oryza_sativa_subsp._japonica proteins in UniProt plus OsWRKY7-SR. Tandem mass spectra were searched against uniprot database. Two missed tryptic cleavages are allowed. Mass error was set to 10 ppm for precursor ions and 0.02 Da for fragment ions. Peptide confidence was set at high, and peptide ion score was set > 20.

**Accession numbers**

Sequences of genes and vectors used in this study can be found in the GenBank library under the following accession numbers: *OsWRKY7* (Os05g0537100), *OsWRKY10* (Os01g0186000), *OsWRKY26* (Os01g0714800), *OsWRKY67* (Os05g0183100), *OsWRKY3* (Os03g0758000), *OsWRKY5* (Os05g0137500), *OsWRKY14* (Os01g0730700), *OsPR1a* (Os07g0129200), *OsPR1b* (Os01g0382000), *OsPR5* (Os12g0628600), *OsPR10a* (Os12g0555500), *OsRbohB* (Os09g0438000), *OsRohbE* (Os08g0453700), *OsActin1* (Os11g0163100), *H2B* (AT5G22880),

Reference

Federici, F., Dupuy, L., Laplaze, L., Heisler, M. and Haseloff, J. (2012) Integrated genetic and computation methods for in planta cytometry. *Nature methods* **9**, 483-485.

Ma, X., Zhang, Q., Zhu, Q., Liu, W., Chen, Y., Qiu, R., Wang, B., Yang, Z., Li, H., Lin, Y., Xie, Y., Shen, R., Chen, S., Wang, Z., Guo, J., Chen, L., Zhao, X., Dong, Z. and Liu, Y.G. (2015) A Robust CRISPR/Cas9 System for Convenient, High-Efficiency Multiplex Genome Editing in Monocot and Dicot Plants. *Mol Plant* **8**, 1274-1284.

Pfaffl, M.W. (2001) A new mathematical model for relative quantification in real-time RT-PCR. *Nucleic Acids Res* **29**, e45.

Zhou, J., Li, D., Zheng, C., Xu, R., Zheng, E., Yang, Y., Chen, Y., Yu, C., Yan, C., Chen, J. and Wang, X. (2020) Targeted Transgene Expression in Rice Using a Callus Strong Promoter for Selectable Marker Gene Control. *Frontiers in plant science* **11**, 602680.
